# Circular RNAs: Their Role in the Pathogenesis and Orchestration of Breast Cancer

**DOI:** 10.3389/fcell.2021.647736

**Published:** 2021-03-11

**Authors:** Xiao He, Tao Xu, Weijie Hu, Yufang Tan, Dawei Wang, Yichen Wang, Chongru Zhao, Yi Yi, Mingchen Xiong, Wenchang Lv, Min Wu, Xingrui Li, Yiping Wu, Qi Zhang

**Affiliations:** ^1^Department of Plastic Surgery, Tongji Hospital, Tongji Medical College, Huazhong University of Science and Technology, Wuhan, China; ^2^Department of Thyroid and Breast Surgery, Tongji Hospital, Tongji Medical College, Huazhong University of Science and Technology, Wuhan, China

**Keywords:** breast cancer, circular RNA, microRNA sponge, metastasis, diagnosis, drug resistance

## Abstract

As one of the most frequently occurring malignancies in women, breast cancer (BC) is still an enormous threat to women all over the world. The high mortality rates in BC patients are associated with BC recurrence, metastatic progression to distant organs, and therapeutic resistance. Circular RNAs (circRNAs), belonging to the non-coding RNAs (ncRNAs), are connected end to end to form covalently closed single-chain circular molecules. CircRNAs are widely found in different species and a variety of human cells, with the features of diversity, evolutionary conservation, stability, and specificity. CircRNAs are emerging important participators in multiple diseases, including cardiovascular disease, inflammation, and cancer. Recent studies have shown that circRNAs are involved in BC progress by regulating gene expression at the transcriptional or post-transcriptional level *via* binding to miRNAs then inhibiting their function, suggesting that circRNAs may be potential targets for early diagnosis, treatment, and prognosis of BC. Herein, in this article, we have reviewed and summarized the current studies about the biogenesis, features, and functions of circRNAs. More importantly, we emphatically elucidate the pivotal functions and mechanisms of circRNAs in BC growth, metastasis, diagnosis, and drug resistance. Deciphering the complex networks, especially the circRNA-miRNA target gene axis, will endow huge potentials in developing therapeutic strategies for combating BC.

## Introduction

Breast cancer (BC) is the most frequently occurring malignancy and the leading cause of cancer-related death among women worldwide. In the last decade, despite therapies, such as surgery, radiation, chemotherapy, endocrine therapy, targeted therapy, and immunotherapy, have achieved certain curative effect, the incidence rates for BC have been rising over the world (Trimboli et al., 2020). The high mortality rates in BC patients are associated with recurrence and metastatic progression to distant organs, including the bone, lung, brain, and liver. In addition, as the resistance of disseminated tumor cells to existing therapeutic agents, the resistance of therapies is also emerging as an urgent issue (Jiang et al., 2020). Therefore, to improve the BC diagnosis and therapy with efficiency, it is imperative to explore the molecular mechanisms of BC pathogenesis.

Nowadays, genetic mutations and epigenetic modifications are playing increasingly significant roles in the BC process. Epigenetic modifications mainly include DNA methylation, histone modifications, and non-coding RNA (ncRNA) (Wu et al., 2020). It is well-established that the occurrence and development of BC are intricately regulated by a variety of ncRNAs, including long non-coding RNA (lncRNA), microRNA (miRNA), and circular RNA (circRNA) (Xu et al., 2020). Notably, as a member of short endogenous ncRNA, circRNA is predominantly derived from gene exons with a closed-loop structure (Xie et al., 2020). This circular structure differs from linear RNA in that it lacks the exposed 5′-terminal cap and 3′-polyadenylate tail structure, reflecting the ability of the circRNA to tolerate exonuclease digestion. CircRNAs are widely found in different species and a variety of human cells, with the features of diversity, evolutionary conservation, stability, and specificity. CircRNAs have been proven to possess a wide range of biological effects, especially functioning to sponge miRNAs as competing endogenous RNA (ceRNA) to offset the impact of miRNAs (Han et al., 2020). In recent years, the chelating protein or transcription regulation of circRNA function has also been gradually reported and attracted attention. Generally speaking, circRNAs play an important role in gene regulation, and their changes in abundance are involved in shaping many disease initiations and processes.

With the progress of the research, it is gradually recognized that circRNA dysregulation is closely related to the occurrence, initiation, and progression of multiple cancer types. It is found that circRNA expression is dysregulated in BC tissue and is closely related to the occurrence and development of BC. For example, Wang J. et al. (2019) identified a total of 480 dysregulated circRNAs in exosomes from metastatic patients compared with localized patients, and these dysregulated circRNAs were enriched in eight pathways. Innovative and novel bioinformatics technologies have revealed the ceRNA crosstalk of circRNAs. Shi et al. (2018) collected four paired BC samples for the human circRNA array to construct a genome-wide circRNA profile of BC. The findings showed that 715 circRNAs were upregulated and 440 circRNAs were downregulated, while hsa_circRNA_005230 was upregulated 12.2-fold and hsa_circRNA_406225 was downregulated 12.4-fold. Zhao et al. (2019) selected a total of seven differentially expressed circRNAs, 27 differentially expressed miRNAs, and 102 differentially expressed mRNAs for the construction of the ceRNA network of BC. Additionally, the hsa_circ_0000519 was involved in the circRNA-miRNA-hub gene network in the pathogenesis of BC. These findings have driven circRNAs as a potential target for improving BC treatment. At the same moment, circRNAs are also regarded to be diagnosis and prognosis biomarkers.

Based on the above, the presentation of circRNA in BC is complex and diverse, and is the key point for BC diagnosis and therapy. Herein, this article has reviewed the recent existing studies on the biogenesis, feature, and function classification of circRNAs. More importantly, it focuses on the expression features, impacts, and mechanisms of the reported circRNAs in BC growth and metastasis. It also emphasizes the diagnostic value of circRNAs in BC. The in-depth understanding of the circRNA mechanisms in BC development will provide better insight into circRNA-associated tumorigenesis and screen novel strategies for combating BC.

## Biogenesis and Feature

Since Sanger et al. (1976) firstly discovered that viroids were composed of covalently closed circRNAs in 1976, a variety of circRNAs have been found in different species such as drosophila, mouse, nematode, and human. With the advancement of high-throughput sequencing technology, emerging evidence indicates that circRNAs are widespread in mammals and their functions have been further explored (Qu et al., 2015). It is gradually recognized that circRNAs are closely related to the occurrence and development of diabetes, hypertension, myocardial infarction, tumor, ischemic attack, osteoporosis, and other diseases (Fang et al., 2019). Based on their compositions, circRNAs can be classified into three types: exonic circRNAs (ecircRNAs), circular intronic RNAs (ciRNAs), and exon-intronic circRNAs (EIciRNAs). Based on their positions and their adjacent mRNAs, circRNAs can be classified as ecircRNAs, intronic circRNAs (ciRNAs), antisense circRNAs, sense overlapping circRNAs, and intergenic circRNAs (Li et al., 2019).

**Figure 1:**
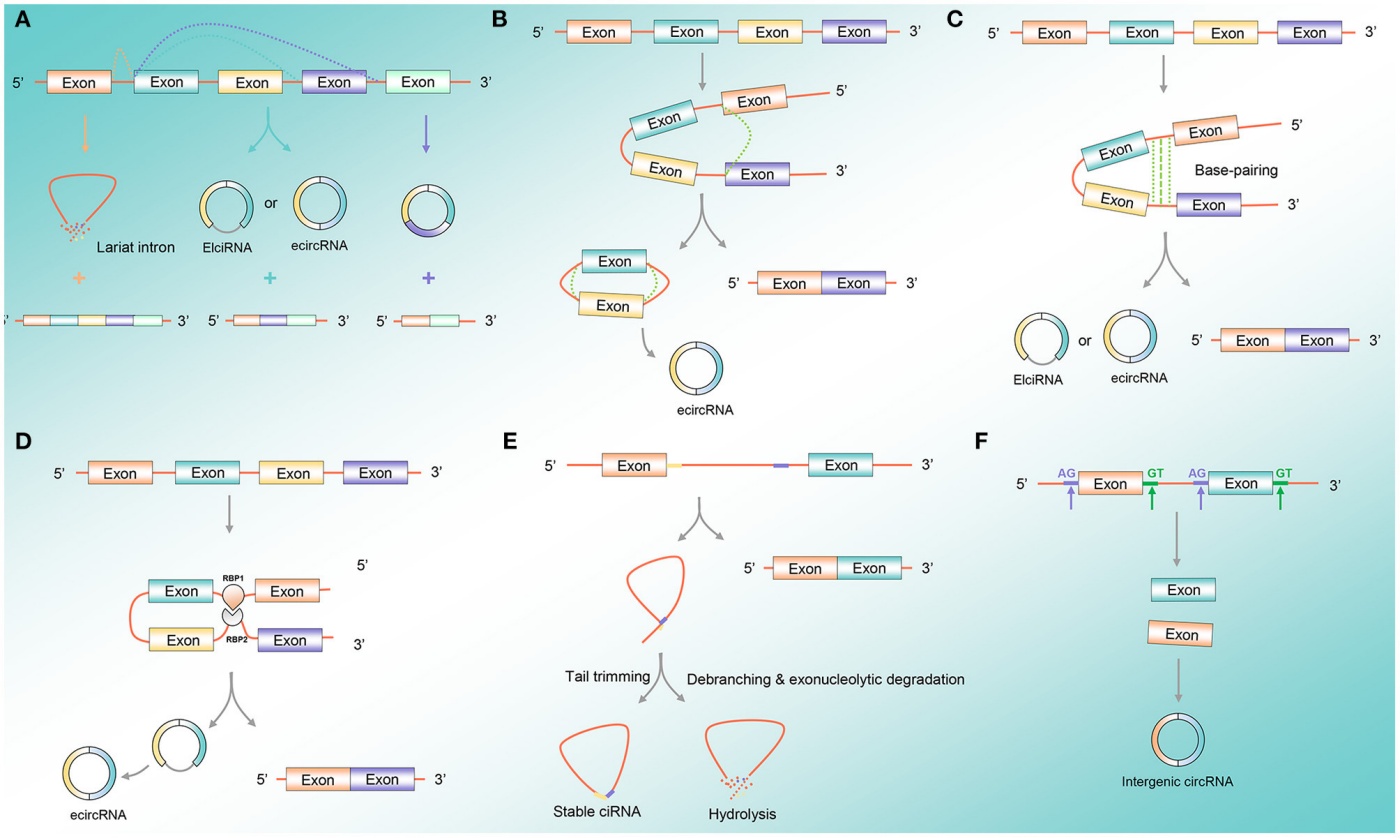
CircRNA formation pattern. **(A)** Variable cyclization mode: circRNA formation is a variable cyclization mode to generate different types of circRNAs according to different splice sites. **(B)** Exon skipping: the pre-mRNA is partially folded during the transcription process, so that the 3′-SD of the downstream exon is connected to the 5′-SA of the upstream exon, causing the exon to skip and form an RNA lariat containing both exons and introns. **(C)** Direct back-splicing: direct back-splicing is the main form of ecircRNA and EIciRNA production, where the flanking intron complementary sequences of pre-mRNAs form a lasso by direct base pairing. **(D)** RBP-dependent circRNA formation: RBPs bind to the sequence motifs of upstream and downstream in introns on both sides of the exon and act as the bridge to draw the distance between the flanking introns to form circRNA. **(E)** CiRNA formation pattern: the pre-mRNA can be spliced into an RNA lasso containing a 2′,5′-phosphodiester, resulted in the formation of ciRNAs after cutting off the 3′-tail. **(F)** Intergenic circRNA formation pattern: the circRNA contains two intronic circRNA fragments flanked by GT-AG splicing signals, acting as the splice donor and splice acceptor of the circular junction to finally form an intergenic circRNA. Abbreviations: circRNA, circular RNA; ecircRNA, exonic circRNA; EIciRNA, exon-intronic circRNA; RBP, RNA-binding protein; ciRNA, circular intronic RNA.

CircRNA formation is a variable cyclization mode to generate different types of circRNAs according to different splice sites, mainly including three mechanisms that permit the back-splicing reaction: exon skipping, direct back-splicing, and dimerization of RNA-binding proteins (RBPs) (Tran et al., 2020) (Figure 1A). Exon skipping is also known as lariat-driven circularization. The pre-mRNA is partially folded in the transcription process, making the 3′-SD of the downstream exon connect with the 5′-SA of the upstream exon, resulting in the exon skipping and forming an RNA lariat containing both exons and introns. The ecircRNAs are further formed with the removal of introns (Geng et al., 2018) (Figure 1B). Direct back-splicing is the main form of ecircRNA and EIciRNA production, in which the flanking intron complementary sequences of pre-mRNAs form a lasso by direct base pairing. The ecircRNAs or EIciRNAs are formed through removing or retaining introns (Figure 1C). RBP-dependent circRNA formation: RBPs bind to the sequence motifs of upstream and downstream in introns on both sides of the exon and act as the bridge to draw the distance between the flanking introns to form circRNA (Figure 1D). The formation of ciRNAs: the pre-mRNA can be spliced into an RNA lasso containing a 2′,5′-phosphodiester, resulted in the formation of ciRNAs after cutting off the 3′-tail (Figure 1E). The intergenic circRNA formation pattern: the circRNA consists of two intronic circRNA fragments flanked by GT-AG splicing signals, which act as the splice donor and splice acceptor of the circular junction to eventually form an intergenic circRNA (Nedoluzhko et al., 2020) (Figure 1F).

CircRNAs are endogenous molecules that display high enrichment, diversity, evolutionary conservation, relative stability, and specificity. CircRNAs also exhibit tissue-specific and developmental phase-specific expression. (i) Diversity: At present, there are many kinds and quantities of circRNAs. CircRNAs are widely expressed in protozoa, fungi, flies, plants, mammals, and humans, and more than 160,000 species of circRNAs have been identified in eukaryotes (Chen et al., 2016). CircRNAs are also present in various body fluids and are widely distributed in tissues and organs. In some special cases, circRNAs are transcribed in a single cell in greater quantities than related linear RNAs, such as mRNAs. (ii) Evolutionary conservation: The expression profiles of circRNAs are highly evolutionary conserved across species not only in mammals but also in flies, plants, and zebrafish, which are relatively far away in evolution (Barrett and Salzman, 2016). The conservation may imply the important role of circRNAs in biological functions. The study of Jeck et al. (2013) discovered that circRNAs were conserved and non-random products of RNA splicing, related to ALU repeats, and involved in regulating gene expression. Venø et al. (2015) also found that approximately 20% of porcine splice sites were involved in circRNA productions between mice and humans with conservative functions. (iii) Stability: The 5′- and 3′-ends are connected end to end to form the covalently closed circular molecule (Hansen et al., 2013), structurally lacking the easily degraded poly-A tail of 3′-end in structure (Greene et al., 2017). Moreover, circRNAs obtain high stability *via* RNA folding (Zhang et al., 2013). Interesting, due to the lack of the 2–5 chain of the RNA lasso characteristic structure, ciRNAs are resistant to RNA debranching enzymes and can exist stably. Thus, circRNAs can avoid RNA degradation pathways. The stability of circRNAs endows their potential to serve as enriched and identifiable ncRNA markers, hence bringing innovative directions in circRNA-based research (Zhou et al., 2019). (iv) Specificity: In disparate cells and tissues, circRNAs possess specific expression profiles, which change spatiotemporally with the development of the cell cycle (Zhang et al., 2015). Importantly, circRNAs differ significantly in normal and pathological tissues.

## Function Classification of circRNAs

**Figure 2:**
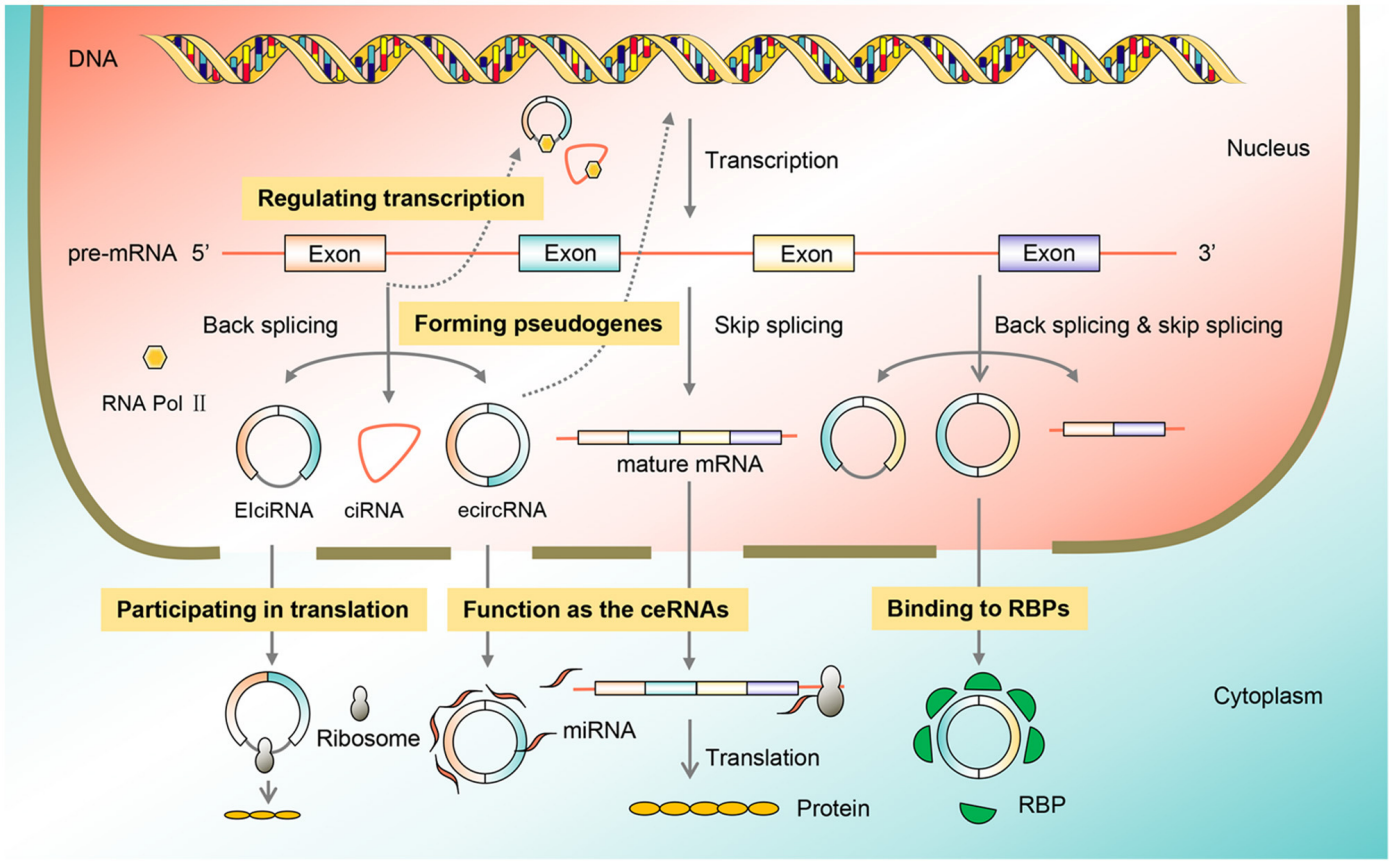
Function classification of circRNAs. CircRNAs are omnipresent in eukaryotic cells, and their functions include acting as the ceRNAs, binding to the RBP, regulating transcription, participating in translation, and forming pseudogenes, thus participating in the process of BC. BC, breast cancer; circRNA, circular RNA; RBP, RNA-binding protein.

CircRNAs are ubiquitous in eukaryotic cells, and their functions are diverse including adsorption of miRNAs, interaction with RBPs, regulation of transcription, involvement in translation, and formation of pseudogenes. In cancer, circRNA function as tumor promoters or suppressors by acting as miRNA sponges (Figure 2).

### Acting as the ceRNAs

The majority of endogenous circRNAs located in the cytoplasm have been identified to have a huge miRNA-binding capacity. miRNAs are transcripts that contained a complementary sequence with target mRNAs, resulting in a substantial alteration of mRNA activity. The most studied function of circRNAs is miRNA sponging, also known as ceRNAs, which can enhance downstream gene expression and regulate cell function and related disease development (Chao et al., 1998). Numerous studies have reported that CDR1as, also called ciRS-7, contained more than 70 conserved miR-7 binding sites (Memczak et al., 2013). The ciRS-7 competitively binds to miR-7 *via* the ceRNA mechanism, affecting miR-7 activity, thus playing a significant role in gastric cancer, colorectal cancer, and other cancer types (Pan et al., 2018). As the most vital circRNA mechanism, ceRNAs are involved in the occurrence and growth of tumors through circRNA axis-associated signaling pathways (Wang et al., 2017).

### Binding to RBPs

RBPs are a class of proteins that bind to RNA and help regulate important processes, such as cell function, cell communication, localization, particularly in the post-transcriptional regulation of RNA. Due to the crucial role in circRNA splicing, processing, folding, stabilization, and localization, RBPs are essential for the generation of circRNAs (Legnini et al., 2017). CircRNAs can bind to different RBPs to exert potential roles such as inhibiting the function of proteins, facilitating the formation of protein complexes, and permitting the interaction between different proteins (Tran et al., 2020). For instance, circ-Foxo3 can form a complex with p21 protein and cyclin-dependent kinases 2 (CDK2), inhibit CDK2 function, block the transformation from the G1 phase to S phase, thereby holding back the proliferation of cancer cells (Cao et al., 2020).

### Regulating Transcription

Some specific circRNAs, including EIciRNAs, intronic circRNAs, and circRNAs composed of exons and introns, are regulators in transcriptional and post-transcriptional levels. EIciRNAs are a type of circRNAs that circulate between exons and introns, and retain between exons, also known as exon-intron circRNA. EIciRNA can modulate the expression of genes by interacting with U1 small nuclear RNA (snRNA). Mechanistically, EIciRNA-U1 snRNA compounds may interact with RNA polymerase II future complex to promote gene expression (Sun et al., 2019).

### Participating in Translation

CircRNAs have long been defined as untranslatable ncRNAs because of their lack of cap structure and poly tail, which are required for linear mRNA translation (Li et al., 2017). Chen and Sarnow (1995) first proposed the hypothesis that circRNA transcripts could be translated *in vitro*. With the in-depth studies, most circRNAs are endogenous and are located primarily in the cytoplasm without translation function, but some circRNAs in the cytoplasm containing start codons, exons, and internal ribosome entry sites (IRES) can bind to initiation factors or ribosomes for translation. As proven by the study of Zhang M. et al. (2018), circ-SHPRH contained the open reading frame (ORF), which could be driven by IRES to translate functional protein SHPRH-146aa, leading to the inhibited proliferation of U251 and U373 glioblastoma cells.

### Forming Pseudogenes

Pseudogenes abound in the human genome and are traditionally regarded as useless “junk genes.” However, recent studies have verified that pseudogenes play a key regulatory role at the level of DNA, RNA, and proteins, thus participating in different physiological and pathophysiological states (Lou et al., 2020). Some studies have indicated that many pseudogenes are de-regulated in cancer, as well as that circRNAs can form pseudogenes through reverse transcription (Ruan et al., 2020). Based on an RNA-sequencing resource from 293 samples including 13 types of cancer and normal tissues, Kalyana-Sundaram et al. (2012) confirmed that at least 33 pseudogenes might all come from circRFWD2. Braicu et al. (2019) verified that a kind of pseudogene RPL13AP17 could act as a tumor suppressor to inhibit the development of lung cancer. The correlation between circRNAs and pseudogenes is not yet fully understood. The role of pseudogene-derived circRNAs in a variety of cancers and the underlying ceRNA mechanism may provide some key clues to identify potential therapeutic targets.

## The Roles and Mechanisms of circRNAs in BC

As a novel class of epigenetic regulators, circRNAs are attracting greater attention and are known to function either as oncogenic or anticancer genes in BC tumor growth and metastasis. In addition, circRNAs have many miRNA binding sites, thereby sponging miRNAs to suppress miRNAs binding to target genes, and subsequently affecting tumor growth (Table 1).

**Table 1:** The dysregulated circRNAs and their mechanisms in breast cancer growth.

**circBase ID (allas)**	**Location**	**Gene symbol**	**Dysregulation**	**miRNA sponge**	**Target gene/pathway**	**Reference**
hsa_circ_0001982	chr15:59323002-59323901	RNF111	Upregulated	miR-143	–	Tang et al., 2017
hsa_circ_0007534	chr17:61869771-61877977	DDX42	Upregulated	miR-593	MUC19	Song and Xiao, 2018
hsa_circ_0008039	chr7:716865-751164	PRKAR1B	Upregulated	miR-432-5p	E2F3	Liu et al., 2018b
hsa_circ_0005230	chr1:172109619-172113577	DNM3OS	Upregulated	miR-618	CBX8	Xu et al., 2018
hsa_circ_0052112	chr19:53158813-53164096	ZNF83	Upregulated	miR-125a-5p	–	Zhang et al., 2018a
hsa_circ_0006528	chr5:145197456-145205763	PRELID2	Upregulated	miR-7-5p	Raf1 and MAPK/ERK	Gao et al., 2017, 2019
hsa_circ_0007331	chr3:195101737-195112876	ACAP2	Upregulated	miR-29a/b-3p	COL5A1	Zhao et al., 2020
hsa_circ_0000519	chr14:20811436-20811534	RPPH1	Upregulated	miR-328-3p	COL1A1	Liu T. et al., 2020
hsa_circ_0005239	chr10:117849251-117856275	GFRA1	Upregulated	miR-34a	GFRA1	He et al., 2017
hsa_circ_0058514	chr2:228356262-228389631	AGFG1	Upregulated	miR-195-5p	CCNE1	Yang R. et al., 2019
hsa_circ_0056289	chr2:121992796-122007223	TFCP2L1	Upregulated	miR-7	PAK1	Wang Q. et al., 2019
hsa_circ_0005320	chr17:75398140-75398785	SEPT9	Upregulated	miR-637	LIF	Zheng et al., 2020
hsa_circ_0001098	chr2:215632205-215646233	BARD1	Downregulated	miR-3942-3p	BARD1	Zhao et al., 2018
hsa_circ_0001184	chr21:34797903-34797990	IFNGR2	Downregulated	miR-449a	Notch1	Wang H. et al., 2018
hsa_circ_0087378	chr9:87356806-87367000	NTRK2	Downregulated	miR-1260b	SFRP1	Yuan et al., 2019
circ-ITCH	Chromosome 20q11.22	ITCH	Downregulated	miR-214 and miR-17	Wnt/β-catenin	Wang S. T. et al., 2019
hsa_circ_0001283	chr3:39108038-39130809	WDR48	Downregulated	miR-187	HIPK3	Hu Y. et al., 2020
hsa_circ_0004771	chr21:16386664-16415895	NRIP1	Upregulated	miR-653	ZEB2	Xie et al., 2019
hsa_circ_0000677	chr16:16101672-16162159	ABCC1	Upregulated	–	PI3K-AKT	Xu J. et al., 2019
hsa_circ_0069718	chr4:52729602-52765544	DCUN1D4	Upregulated	–	Wnt/β-catenin	Zhang et al., 2019
hsa_circ_0038632	chr16:23691404-23701688	PLK1	Upregulated	miR-296-5p	PLK1	Kong et al., 2019
CDR1-AS	–	–	Downregulated	miR-7	–	Uhr et al., 2018
circ-UBE2D2	–	–	Downregulated	miR-1236 and miR-1287	–	Wang Y. et al., 2019

“*–” Not applicable. circRNA, circular RNA; miRNA, microRNA*.

### CircRNAs in BC Growth

#### The Promoting Effect of circRNAs in BC Growth

As circRNA CDR1-AS was able to specifically sponge human miR-7, Uhr et al. (2018) further emphasized that CDR1-AS was differentially expressed among the BC cell line subtypes with higher CDR1-AS levels in the luminal and normal-like subtypes. Tang et al. (2017) screened the circRNA expression profiles in BC tissue using circRNA microarray analysis and found that hsa_circ_0001982 was overexpressed in BC tissue and cell lines. Through dual-luciferase reporter assay, miR-143 was verified to be the target of hsa_circ_0001982. The inhibition of hsa_circ_0001982 could restrain BC cell proliferation and invasion and promote apoptosis by targeting miR-143. Song and Xiao (2018) observed the markedly upregulated expression of hsa_circ_0007534 in BC tissues and cell lines. Hsa_circ_0007534 knockdown could suppress BC cell proliferation, colony formation, and invasion and induce apoptosis in BC cells *via* acting as a miR-593 sponge to increase MUC19 expression. Xie et al. (2019) performed high-throughput circRNA sequencing to detect the differentially expressed circRNAs and confirmed that hsa_circ_0004771 and Zinc finger E-box-binding homeobox 2 (ZEB2) expression levels were upregulated and positively correlated in BC tumor tissues. Mechanistically, hsa_circ_0004771 functioned as a sponge of miR-653 and then downregulated its expression. miR-653 could inhibit ZEB2 expression *via* binding to its 3′-UTR. Intriguingly, the knockdown of hsa_circ_0004771 and ZEB2 served as equally authentic of miR-653 mimics to induce growth inhibition and apoptosis in BC cells. Liu et al. (2018b) distinguished a new circRNA hsa_circ_0008039 with a high expression level in BC tissues. Hsa_circ_0008039 knockdown distinctly inhibited the proliferation and migration in BC. Mechanistic analyses indicated that hsa_circ_0008039 acted as a sponge of miR-432-5p to improve E2F3 expression.

Xu et al. (2018) confirmed that circ_0005230 was upregulated in BC tissue specimens and cell lines, which was associated with adverse phenotypes in BC patients. Data *in vitro* and *in vivo* verified the promoting effect of circ_0005230 on cell growth, cell migratory, and invasive capacities. Regarding the mechanism, circ_0005230 could play a carcinogenic role in BC by sponging miR-618 to increase CBX8 expression. Hsa_circ_0052112, which was significantly higher in MDA-MB-231 cells than that in MCF-7 cells, might promote the migration and invasion of BC cells *via* directly sponging to miR-125a-5p and possibly upregulating ZNF83 expression (Zhang et al., 2018b). Xu J. et al. (2019) found that hsa_circ_001569 expression was significantly upregulated in both BC tissues and cell lines while hsa_circ_001569 might contribute to the progression of BC by modulating the PI3K-AKT pathway. Karedath et al. (2019) identified and characterized a circRNA derived from the ANKRD12 gene, termed as circANKRD12, which was abundantly expressed in breast and ovarian cancer cells. Manipulating the levels of circANKRD12 induced a strong phenotypic change by significantly regulating cell cycle, invasion, and migration and altering the metabolism in cancer cells (Karedath et al., 2019). Circ_0006528 could facilitate DNA synthesis and cell proliferation, invasion, and migration in BC cells. For the mechanism investigation, circ_0006528 could sponge miR-7-5p to promote Raf1 expression, which activated the MAPK/ERK signaling pathway (Gao et al., 2019). Zhao et al. (2020) applied bioinformatics analysis to finding that circACAP2 expression was elevated in BC tissues and could lead to the malignant phenotype of BC cells. Mechanistically, circACAP2 induced BC proliferation and motility by sponging miR-29a/b-3p and regulating COL5A1. Wang Y. et al. (2019) found that circ-UBE2D2 was a high expressional biomarker closely associated with aggressive clinical features and dismal prognosis. Circ-UBE2D2 could significantly suppress the proliferation, migration, and invasion of BC cells by binding to miR-1236 and miR-1287, thus modulating the expression of their respective target genes. Besides, the delivery of cholesterol-conjugated si-circ-UBE2D2 oligonucleotides significantly repressed tumor growth *in vivo*. Liu T. et al. (2020) analyzed that higher expression of hsa_circRNA_002178 led to the worse prognosis in BC by competitively harboring miR-328-3p and then increasing COL1A1 expression. *In vivo* experiments further demonstrated the suppression of tumor growth and inflammation by inhibiting hsa_circRNA_002178 or upregulating miR-328-3p.

Some specific circRNAs are tumor promoters in the malignant transformation of triple-negative breast cancer (TNBC). He et al. (2017) conducted circRNA microarrays and observed that circGFRA1 was abnormally upregulated in TNBC. Upregulated circGFRA1 was associated with poorer survival *via* sponging miR-34a to modulate GFRA1 expression. CircRNA_069718 was a tumor oncogenic circRNA in TNBC progression. CircRNA_069718 inhibition reduced the expression levels of Wnt/β-catenin pathway-related genes, including β-catenin, c-myc, and cyclin D1 (Zhang et al., 2019). In four paired TNBC tissues and para-cancerous tissues using RNA sequencing, Yang R. et al. (2019) found a total of 354 circRNAs were differentially expressed with 47 upregulated and 307 downregulated expressions. Among them, circAGFG1 was the most upregulated circRNA spliced from AGFG1 and was correlated with clinical stage, pathological grade, and poor prognosis of TNBC patients. CircAGFG1 could act as a ceRNA for sponging miR-195-5p to alleviate the inhibition of miR-195-5p on CCNE1, thus accelerating the cell division. CircPLK1 was significantly upregulated in TNBC and associated with worse survivals. The knockdown of circPLK1 efficiently inhibited the cell growth and invasion *in vitro* as well as tumor occurrence and metastasis *in vivo*. Therefore, the ceRNA mechanism of the circPLK1-miR-296-5p-PLK1 axis regulated the tumor progression in TNBC (Kong et al., 2019). Wang Q. et al. (2019) conducted a human circRNA microarray in three pairs of TNBC tissues and adjacent non-cancerous tissues to screen circRNA expression patterns in TNBC. The results confirmed that circ-TFCP2L1 was markedly upregulated in TNBC tissues and cells and led to the shorter disease-free survival for TNBC patients. Circ-TFCP2L1 induced the proliferation and migration of TNBC by sponging miR-7 so as to decrease PAK1 expression. Mechanistic studies revealed that circSEPT9 could sponge miR-637 and activate the leukemia inhibitory factor (LIF)/STAT3 signaling pathway involved in TNBC progression to modulate the expression of LIF (Zheng et al., 2020). In addition, E2F1 and EIF4A3 might improve the biogenesis of circSEPT9.

#### The Inhibiting Effect of circRNAs in BC Growth

Breast cancer stem cells (BCSCs) are defined as a small subset of cancer cells with the properties of increasing self-renewal, multilineage differentiation, and tumor maintenance, all of which are considered contributions to the BC progression, metastasis, and recurrence. It was of note that Yan et al. (2017) proposed the work to prove the evidence of the circRNA profiles and circRNA/miRNA interplays in BCSCs. They found 27 aberrantly expressed circRNAs, of which circVRK1 could suppress BCSC proliferation and self-renewal capacity. In BC cell MCF-7 with the treatment of tetrachlorodibenzo-p-dioxin (TCDD), circRNA_BARD1 was upregulated to suppress cell proliferation, block cell cycle, and induce cell apoptosis (Zhao et al., 2018). Moreover, circRNA_BARD1 was overexpressed by TCDD and then suppressed BC tumorigenesis *via* miR-3942-3p/BARD1 axis both *in vivo* and *in vitro*. Wang H. et al. (2018) conducted circRNA microarray analysis to investigate abnormally expressed circRNAs in BC tissue and confirmed that circRNA-000911 was downregulated in BC cells. CircRNA-000911 could restrain cell proliferation, migration, and invasion and facilitate the apoptosis of BC cells *via* capturing miR-449a to improve Notch1 expression and inhibiting nuclear factor-κB (NF-κB) signaling. By systematically profiling circRNA expression patterns, Yuan et al. (2019) observed 3,653 differentially expressed circRNAs in estrogen receptor (ER)-positive BC compared with adjacent non-tumor tissues. Among them, has_circ_0072304, has_circ_0087378, and has_circ_0087377 were verified to be downregulated in ER-positive BC. Besides, the has_circ_0087378-miR-1260b-SFRP1 axis was considered to be a key regulatory pathway in BC tumorigenesis (Yuan et al., 2019). Wang S. T. et al. (2019) reported that circ-ITCH was significantly downregulated in TNBC and correlated with poor prognosis. Furthermore, circ-ITCH could bind miR-214 and miR-17 to increase its expression of ITCH linear isoform, thereby inactivating Wnt/β-catenin signaling and then blocking the expression of downstream genes to inhibit the proliferation of BC cells (Wang S. T. et al., 2019). Sang et al. (2019) conducted RNA sequencing on a large number of clinical samples and screened their circRNA expression profiles and demonstrated that hsa_circ_0025202 was significantly decreased in BC tissues and could restrain the proliferation and metastasis of BC cells. Hu Y. et al. (2020) observed that the circRNA-0001283 expression was downregulated in BC tissue samples. CircRNA-0001283 ectopic expression remarkably suppressed BC cell viability and invasion and promoted apoptosis in BC cells *via* decreasing the expression of miR-187 and then positively regulating HIPK3 expression, which might provide targets for developing novel therapeutic strategies for BC.

### CircRNAs in BC Metastasis

Metastasis is the result of multiple steps, including local infiltration of tumor cells through the surrounding extracellular matrix (ECM) and stromal cell layer, interpolation, and survival in the circulation, extravasation, and seeding to distant places and the beginning of metastasis and colonization. CircRNAs play vital roles in metastasis and cancer progression (Table 2). For instance, Li Z. et al. (2020) indicated that a total of 4,370 circRNAs in general expression profiling, with 2,375 upregulated circRNAs and 1,995 downregulated circRNAs in these BC/adjacent tissues. Hsa_circ_0069094, hsa_circ_0079876, hsa_circ_0017650, and hsa_circ_0017526 were promising candidate targets, as they were associated with tumor-node metastasis (TNM) staging, lymph node infiltration, and Ki67.

**Table 2:** The dysregulated circRNAs and their mechanisms in breast cancer metastasis.

**circBase ID (allas)**	**Location**	**Gene symbol**	**Dysregulation**	**miRNA sponge**	**Target gene/pathway**	**Reference**
hsa_circ_0011946	chr1:41578954-41618413	SCMH1	Upregulated	miR-26a/b	RFC3	Zhou et al., 2018
hsa_circ_0072995	chr5:73069679-73076570	RGNEF	Downregulated	miR-30c-2-3p	–	Zhang et al., 2018b
hsa_circ_0061825	chr21:43782390-43786644	TFF1	Upregulated	miR-326	TFF1	Pan et al., 2020
hsa_circ_0007294	chr12:100166699-100175875	ANKS1B	Upregulated	miR-148a-3p and miR-152-3p	TGF-β1/Smad	Zeng et al., 2018
hsa_circ_0005505	chr12:66597490-66622150	IRAK3	Upregulated	miR-3607	FOXC1	Wu et al., 2018
hsa_circ_0000247	chr10:74474868-74475660	MCU	Upregulated	miRNA let-7 family members	MCY and HMGA2 and CCND1	Song et al., 2020
hsa_circ_0001839	chr9:6880011-6893232	KDM4C	Downregulated	miR-548p	PBLD	Liang et al., 2019b
hsa_circ_0072309	chr5:38523520-38530768	LIFR	Downregulated	miR-492	CD147	Yan et al., 2019
hsa_circ_103809	Chromosome 5p13.3	ZFR	Downregulated	miR-532-3p	EMT	Liu M. et al., 2020
circEHMT1	Chromosome 9	EHMT1	Downregulated	miR-1233-3p	KLF4	Lu et al., 2020
hsa_circ_0002018	chr19:3449011-3452664	NFIC	Downregulated	miR-658	UPK1A	Xu G. et al., 2020
circDENND4C	–	–	Upregulated	miR-200b and miR-200c	–	Ren et al., 2019
hsa_circ_0001944	chrX:130883333-130928494	TCONS_l2_00030860	Upregulated	miR-509	RhoC-TNF-α	Fu et al., 2018
hsa_circ_21439	–	–	Upregulated	miR-26b and miR-335	EphA2 and SOX4	Lin et al., 2019
hsa_circ_0006220	chr17:35800605-35800763	TADA2A	Downregulated	miR-203a-3p	SOCS3	Xu J.-Z. et al., 2019
hsa_circ_0089105	chr9:133352257-133355836	ASS1	Downregulated	miR-4443	ASS1	Hou et al., 2019
hsa_circ_11783	–	–	Downregulated	miR-365-5p	AKT	Lin et al., 2019

“*–” Not applicable. circRNA, circular RNA; miRNA, microRNA*.

#### The Promoting Effect of circRNAs in BC Metastasis

Elucidation of the underlying mechanisms of circRNA-promoting metastasis is desperately needed to offer novel therapeutic strategies for patients with metastatic BC. Cancer metastasis is a complex and multistep process. Zhou et al. (2018) utilized high-throughput sequencing to uncover the 152 differentially expressed circRNAs in BC tissues and cell lines and found that hsa_circ_0011946 was significantly upregulated. Whereas, the inhibition of hsa_circ_0011946 significantly suppressed the migration and invasion of MCF-7 cells. Possibly, hsa_circ_0011946 sponged miR-26a/b to directly target replication factor C subunit 3 (RFC3) and knockdown of hsa_circ_0011946 could inhibit RFC3 mRNA and protein expression thus promoting cell migration and invasion, as confirmed by bioinformatics analysis and validation. Hsa_circ_0072995 was generated from the ARHGEF28 gene and also might be a novel biomarker for BC (Zhang et al., 2018b). Hsa_circ_0072995 could promote BC cell migration and invasion by capturing miR-30c-2-3p. Besides, the overexpression of hsa_circ_0072995 upregulated ARHGEF28 expression, which activated the activity of FAK and RhoC, enhancing migration and invasion capability of BC cells. Zhang et al. (2019) investigated the potential regulatory mechanism of circRNA_069718 in BC and found that circRNA_069718 expression was increased in BC tissues and cell lines and its high expression was significantly correlated with advanced TNM, lymph node metastasis, and poor overall survival ability in BC patients. Interestingly, circDENND4C was a HIF1A-associated circRNA and was highly expressed in hypoxia conditions to promote BC cell proliferation. Silencing circDENND4C suppressed glycolysis, migration, and invasion in BC cells under hypoxia possibly by promoting the expression miR-200b and miR-200c (Ren et al., 2019). This work highlighted circDENND4C as a novel prognostic and therapeutic target for BC. Pan et al. (2020) observed that circ-TFF1 and TFF1 were both overexpressed and positively correlated with each other in BC. Circ-TFF1 could promote BC cell proliferation, migration, invasion, and epithelial-mesenchymal transition (EMT) *in vitro* and facilitate tumor growth *in vivo via* sponging miR-326 to increase TFF1 expression.

Some circRNAs possess a promoting impact on BC lung metastasis. Zeng et al. (2018) reported that circANKS1B feedback loop promoted BC invasion and metastasis both *in vitro* and mouse lung metastasis model by inducing EMT, with no effect on BC growth. Mechanistically, circANKS1B abundantly sponged miR-148a-3p and miR-152-3p to increase the expression of USF1, which could transcriptionally upregulate TGF-β1 expression, thereby activating TGF-β1/Smad signaling for enhancing EMT (Zeng et al., 2018). Wu et al. (2018) verified an oncogenic circRNA derived from the IRAK3 gene locus, named circIRAK3, which was closely related to the poor prognosis of BC patients with recurrence to distant organs. Besides, circIRAK3 was significantly upregulated in metastatic BC cells and could promote cell migration, invasion, and lung metastasis by the sponge of miR-3607 to regulate FOXC1 expression, thus triggering a positive-feedback loop. CircHMCU exerted oncogenic functions including promoting the migration and invasion, *via* efficiently harboring the let-7 family to increase the expression of MYC, HMGA2, and CCND1. Further *in vivo* studies showed the overexpression of circHMCU resulted in the rapid proliferation and lung metastasis of BC (Song et al., 2020).

Breast cancer brain metastases (BCBM) often cause neurological impairments by affecting cognitive and sensory functions, which occupy a proportion of diagnosed metastatic BC and confer an extremely poor prognosis. Identifying the mechanism of key circRNAs of BCBM is a prerequisite for unlocking BCBM to predict, diagnose, and future innovative treatments. Fu et al. (2018) performed RNA-sequencing to identify the circRNA expression profiles of BCBM cell line 231-BR in comparison with non-specific metastatic MDA-MB-231. The result verified that 215 upregulated and 191 downregulated circRNAs, among which 90% differentially expressed circRNAs were generated from the exonic regions of host genes and demonstrated that the most enriched pathways of the differentially expressed circRNAs were related to the RNA degradation and axon guidance. It hypothesized that has_circ_0001944 might be involved in BCBM through sponging up miR-509 and interfering with its downstream targets, thereby forming the has_circ_0001944/miR-509/RhoC-TNF-α axis (Fu et al., 2018). Lin et al. (2019) reported that hsa_circ_21439 was verified to be the most significantly upregulated in breast cancer liver metastases (BCLM) tissues and cells. The has_circ_21439 would regulate the process of BCLM through has_circ_21439/miR-26b/EphA2 axis and/or has_circ_21439/miR-335/SOX4 axis.

#### The Inhibiting Effect of CircRNAs in BC Metastasis

By screening the circRNA profiles in a large cohort of BC patients, Xu J.-Z. et al. (2019) distinguished 235 differentially expressed circRNAs including the two differentially expressed circTADA2As, circTADA2A-E6, and circTADA2A-E5/E6 in BC. Especially, circTADA2A-E6 possessed the tumor-suppressor capability *in vitro* through sponging a miR-203a-3p to restore the expression of SOCS3, resulting in a less aggressive oncogenic phenotype. Hou et al. (2019) confirmed that circASS1 was significantly downregulated in highly aggressive BC cell line MDA-MB-231 cells compared with MCF-7. CircASS1 suppressed invasion and migration of BC cells by harboring miR-4443 and upregulating the ASS1 expression, implying the tumor-suppressive role of circASS1 in BC metastasis. Liang et al. (2019a) identified a fresh circBMPR2, which was significantly reduced in human BC tissues with metastasis and was negatively associated with the motility of BC. Functionally, the circBMPR2 knockdown effectively enhanced cell proliferation, migration, and invasion (Liang et al., 2019a). In the following studies, they further characterized a novel KDM4C gene-derived circRNA circKDM4C which was decreased in BC tissues of a cohort of 219 BC patients (Liang et al., 2019b). The lower circKDM4C expression was associated with poor prognosis and metastasis in BC, showing the tumor suppressor function of circKDM4C in BC metastasis. Yan et al. (2019) analyzed that the downregulated hsa_circ_0072309 significantly suppressed the proliferation, migration, and invasion of BC cells *in vitro*. *In vivo* assays showed that hsa_circ_0072309 ectopic expression inhibited BC growth *via* the hsa_circ_0072309-miR-492 axis. Liu M. et al. (2020) discovered that circRNA_103809 was downregulated in BC tissues, and circRNA_103809 expression level was closely related to the distant metastasis size, TNM stage, HER-2 status, and overall survival time. *In vitro* experiments indicated that circRNA_103809 overexpression could obviously suppress the EMT pathway to inhibit BC cell proliferation and metastasis *via* harboring miR-532-3p.

In the study of lung metastasis, Lu et al. (2020) reported that circEHMT1 was decreased in human BC tissues, and the overexpression of circEHMT1 inhibited lung metastasis of BC *in vitro* and *in vivo*, emphasizing the importance of circEHMT1 in inhibiting BC metastatic potential by reducing MMP2 levels through modulating circEHMT1/miR-1233-3p/KLF4 axis. Xu G. et al. (2020) demonstrated that circNFIC was the most downregulated circRNA in lung metastatic tissues and that low levels of circNFIC were associated with poor outcome of BC by Kaplan-Meier survival analysis. Moreover, both *in vivo* and *in vitro*, circNFIC functioned as a ceRNA to upregulate UPK1A expression by decoying miR-658, possibly resulting in suppressing BC cell proliferation and migration to the lung (Xu G. et al., 2020). In BCLM and cells, Lin et al. (2019) used RNA sequence to identify the altered expression profile of circRNAs, showing that the downregulated hsa_circ_11783 exerted the functions in BCLM by hsa_circ_11783/miR-365-5p/AKT axis by further bioinformatics exploration.

## Diagnostic Value of circRNAs in BC

Given the unique characteristics of expression, stability, and enriched miRNA binding sites, circRNAs have been endowed with great potential as tumor diagnostic biomarkers and therapeutic targets in BC (Greene et al., 2017) (Table 3). These identified circRNA modules provide novel insights into the potentially BC-associated circRNAs and will contribute to the clinical applications of circRNA for BC diagnosis, treatment, and prognosis in the future. Therefore, identifying changes of circRNA expression in body fluids may open a novel avenue for the non-invasive BC diagnosis.

**Table 3:** The dysregulated circRNAs and their values in breast cancer diagnosis.

**circBase ID (allas)**	**Location**	**Gene symbol**	**Dysregulation**	**miRNA sponge**	**Target gene/pathway**	**Clinical value**	**Reference**
hsa_circ_0001846	chr9:33944362-33956144	UBAP2	Upregulated	miR-661	MTA1	Predicting a poor prognosis of TNBC	Wang S. et al., 2018
hsa_circ_0001819	chr8:103372298-103373854	UBR5	Upregulated	miR-4753/6809	BCL11A	Associated with the decreased survival in TNBC	Chen et al., 2018
hsa_circ_0007255	chrX:69549254-69553539	KIF4A	Upregulated	miR-375	KIF4A	Correlated with poorer survival of TNBC	Tang et al., 2019
hsa_circ_0005571	chr19:18285849-18286507	IFI30	Upregulated	miR-520B-3p	CD44	Predicting a poor prognosis of TNBC	Xing et al., 2020
hsa_circ_0009362	chr1:1756835-1770677	GNB1	Upregulated	miR-141-5p	IGF1R	Related to worse clinical features and survival outcomes of TNBC	Liu P. et al., 2020
hsa_circ_0001859	chr9:37086664-37121125	TCONS_l2_00028722	Upregulated	miR-200c-3p	ZEB1/2and ETS1	Related to poorer prognosis of BC patients	Liu et al., 2019
hsa_circ_0008285	chr6:4891946-4892613	CDYL	Upregulated	miR-1275-ATG7	ULK1	Related to shorter survival and poorer clinical response to treatment of BC	Liang et al., 2020
hsa_circ_0000515	chr14:20811305-20811534	RPPH1	Upregulated	miR-296-5p	CXCL10	Related to poor prognosis of BC	Cai et al., 2020
hsa_circ_0000320	chr11:62297840-62298224	AHNAK	Downregulated	miR-421	RASA1	Negatively associated with recurrence-free survival and overall survival as a predictor of TNBC prognosis	Xiao et al., 2019

“*–” Not validated experimentally. circRNA, circular RNA; miRNA, microRNA; BC, breast cancer; TNBC, triple-negative breast cancer*.

### CircRNA as Potential Prognosis Biomarkers in BC

Identification and elucidation of the effective potential diagnostic and prognostic value of circRNA biomarkers are essential for clinical decision-making. Numerous studies have shown that circRNAs present differential expressions in BC and are linked to the pathogenesis and prognosis-related biomarkers. The integration of circRNAs, miRNAs, DNA methylation, and gene expression data to identify sponge circRNAs is crucial for revealing the DNA methylation-mediated regulation of sponge circRNAs in cancer progression. Gu et al. (2019) revealed a complex DNA methylation-mediated regulation of circRNA sponges, and 10 sponge circRNA host genes were potential prognostic biomarkers of BC. Ma et al. (2020) conducted a weighted gene co-expression network analysis with the differentially expressed miRNAs and mRNAs in BC from The Cancer Genome Atlas (TCGA) database to identify the key modules associated with the carcinogenesis of BC. By database co-screening, has_circ_0083373, hsa_circ_0083374, and hsa_circ_0083375 were involved in the pathogenesis and development of BC, mechanistically through regulating DLC1 expression *via* has-mir-511. Yang et al. (2020) used RNA-sequencing, qPCR, and bioinformatic analysis to explore potential mechanisms involving differentially expressed circRNAs in the serum exosomes and tissues of BC patients. Compared with paracancerous normal tissues, hsa-circRNA-0005795 and hsa-circRNA-0088088 were significantly different both in serum exosomes and tissues and might function as ceRNAs with 11 and 8 circRNA-miRNA interactions, respectively (Yang et al., 2020). The overwhelming majority of circRNAs, which had been reported, showed upregulated expression levels in BC. Therefore, the novel circRNA biomarkers are warranted to be discovered for the early detection and prognosis for BC (Tomar et al., 2020).

#### The Upregulated CircRNAs as Prognosis Biomarkers

TNBC is the most aggressive BC subtype, exhibiting a strong proliferative capacity, increased recurrence, and decreased survival rate. Revealing the role of dysregulated circRNAs will be critical for understanding TNBC pathogenesis and improving the prognosis. Wang S. et al. (2018) observed that circ-UBAP2 with high expression was closely correlated with larger tumor size, lymph node metastasis, advanced TNM stage, and unfavorable prognosis. *In vitro* and *in vivo*, circ-UBAP2 could enhance the proliferation and migration of TNBC cells and suppress apoptosis by sponging miRNA-661 to enhance the oncogene MTA1 expression. Likewise, circANKS1B was notably upregulated in TNBC compared with non-TNBC tissues and cell lines, which was closely associated with lymph node metastasis and advanced clinical stage, serving as an independent risk factor for overall survival of BC patients (Zeng et al., 2018). Chen et al. (2018) found that circEPSTI1 was significantly upregulated and a high level of circEPSTI1 was associated with the decreased survival in TNBC patients. Their experiments showed that circEPSTI1 bound to miR-4753 and miR-6809 as the miRNA sponge to enhance BCL11A expression and then inhibited TNBC cell proliferation and promoted apoptosis, thus presuming that circEPSTI1 might be an independent prognostic marker for survival in TNBC patients. Tang et al. (2019) explored the circRNA profiles by circRNA microarray and observed that circKIF4A was overexpressed in BC cells and TNBC tissues. CircKIF4A promoted the proliferation and migration of TNBC cells *via* sponging miR-375 to modulate KIF4A expression. Thus, circKIF4A might act as a prognostic and therapeutic target for TNBC and positively correlated with poorer survival of TNBC. Through quantitative real-time polymerase chain reaction (qRT-PCR) and *in situ* hybridization, Xing et al. (2020) observed that circIFI30 was upregulated in TNBC tissues and could remarkably enhance TNBC cell malignant biological properties by acting as the miR-520b-3p sponge to improve CD44 expression. The increased circIFI30 was positively associated with the clinical TNM stage, pathological grade, and poor prognosis of TNBC patients, thus circIFI30 could be a new diagnostic/prognostic marker and therapeutic target for TNBC patients (Xing et al., 2020). Liu P. et al. (2020) characterized circGNB1 *via* analyzing the circRNA microarray profiling and found that circGNB1 was overexpressed in TNBC cell lines. Knockdown of circGNB1 obviously inhibited cell proliferation, migration, and tumor growth *via* the circGNB1-miR-141-5p-IGF1R axis. Consequently, circGNB1 might be potential for BC prognosis as that high circGNB1 expression was related to worse clinical features and survival outcomes (Liu P. et al., 2020). Zheng et al. (2020) studied the circRNA expression patterns in four pairs of TNBC tissues and paracancerous normal tissues using RNA-sequencing. The results indicated that circSEPT9 was upregulated in TNBC tissues, which was positively related to the advanced clinical stage and poor prognosis (Zheng et al., 2020).

Liu et al. (2019) systematically investigated the circRNAs and found hsa_circ_001783 was a top-ranked circRNA in both BC cells and tissues. The higher level of hsa_circ_001783 was closely related to heavier tumor burden and poorer prognosis of BC patients. Moreover, hsa_circ_001783 promoted the progression of BC cells by sponging miR-200c-3p to improve the expression of ZEB1, ZEB2, and ETS1. Therefore, hsa_circ_001783 might act as a new prognostic and therapeutic target for BC (Liu et al., 2019). Yang L. et al. (2019) explored a novel increased circ_0103552, with clinical severity and dismal prognosis. Furthermore, circ_0103552 could promote cell growth, clone-forming ability, migration, and invasion and suppress apoptotic cells. Further mechanistic studies revealed that circ_0103552 could directly sponge miR-1236 to perform oncogenic activities in BC cells (Yang L. et al., 2019). Liang et al. (2020) screened autophagy correlated circRNAs in BC tissues with high- and low-autophagic levels by circRNA deep sequencing. The results showed that circCDYL improved the autophagic level in BC cells by the miR-1275-ATG7/ULK1 axis and promoted the malignant progression of BC cells *in vitro* and *in vivo*. Clinically, increased circCDYL in the BC tissues and serum was related to higher tumor burden, shorter survival, and poorer clinical response to treatment (Liang et al., 2020). Cai et al. (2020) gathered in 340 BC tissues and identified hsa_circ_0000515 which was overexpressed and related to the poor prognosis of BC. Knockdown of hsa_circ_0000515 disrupted cell cycle progression, cell proliferation, and invasion, weakened inflammatory response, and decreased the pro-angiogenetic potential of BC cells (Cai et al., 2020). Regarding the mechanism, hsa_circ_0000515 could sponge miR-296-5p to hinder it from inhibiting CXCL10 expression.

Additionally, as circHMCU was upregulated in high metastatic cell lines compared with parental cell lines and in BC tissues compared with normal tissues, circHMCU was related to poor BC prognosis and could be used as a novel biomarker in the diagnosis and prognosis of BC (Song et al., 2020). Gao et al. (2019) analyzed a cohort of 97 patients and observed that circ_0006528 expression was upregulated in BC tissues and was significantly related to advanced TNM stage and poor prognosis. The multivariate assay showed that hsa_circ_001569 expression was an independent prognostic factor for 5-year overall survival (Xu J. et al., 2019). This study indicated that hsa_circ_001569 upregulation was associated with BC lymph-node metastasis, clinical stage, and poor prognosis. Smid et al. (2019) demonstrated an independent circRNA pool of CNOT2, CREBBP, and RERE in primary BC. The circCNOT2 could be detectable in cell-free RNA from plasma and exhibited clinical potential for aromatase inhibitor therapy because circCNOT2 levels were predictive of progression-free survival time to aromatase inhibitor therapy in advanced BC patients.

#### The Downregulated CircRNAs as Prognosis Biomarkers

Circ-ITCH is a tumor repressor and was markedly downregulated in TNBC tissues and cell lines and correlated with poor prognosis (Wang S. T. et al., 2019). Circ-ITCH improved its expression *via* sponging miR-214 and miR-17, which in turn inactivated the Wnt/β-catenin signal and then inhibited the expression of downstream genes. Circ-ITCH was a promising prognostic biomarker in TNBC, and its restoration could well be a successful strategy in TNBC (Wang S. T. et al., 2019). Xiao et al. (2019) confirmed that circAHNAK1 was markedly downregulated in TNBC cell lines, which was negatively associated with recurrence-free survival and overall survival as a predictor of TNBC prognosis. The mechanistic analysis showed that circAHNAK1 inhibited proliferation and metastasis of TNBC *via* acting as the miR-421 sponge to promote RASA1 expression. CircEHMT1 was downregulated in human BC tissues compared with adjacent normal breast tissues (Lu et al., 2020). The AUC value of circEHMT1 was very close to 1, suggesting that circEHMT1 was related to a better prognosis of human BC.

### CircRNA-Based Computational Methods for BC Detection

Increasing evidence indicates that circRNAs are broadly in the occurrence and development of BC. However, due to the complex mechanisms, it is expensive, difficult, and time consuming to discover the new circRNA-disease associations by biological experiments. Therefore, it is an increasingly urgent need to utilize computational methods to predict new circRNA disease associations. Based on existing bioinformatics approaches, Nair et al. (2016) developed a comprehensive workflow called Circ-Seq, which was applied to RNA-sequencing data from BC cell lines. Notably, normal-adjacent tissues in ER-positive subtype had higher numbers of circRNAs than tumor samples in TCGA, and the number of circRNAs in normal-adjacent samples of the ER+ subtype was inversely correlated to the risk-of-relapse proliferation (ROR-P) score for proliferating genes, suggesting that the circRNA frequency might be a marker for cell proliferation in BC. Tarrero et al. (2018) developed a novel computational tool, named CircHunter, to exploit RNA-sequencing data to define a compilation of exonic circRNAs, more accurately. With this mean, a subset of MCF-7 circRNAs was specific to tumor vs. normal tissue, while some circRNAs could distinguish Luminal from other tumor subtypes, thus suggesting that circRNAs could be exploited as novel biomarkers and drug targets for BC. Lei et al. (2018) proposed a new computational path-weighted method Path-Weighed Method for Predicting CircRNA-Disease Associations (PWCDA), showing that circPVT1 could be worked as a miRNA spouse to regulate miRNA by moderating let-7 activity in BC. Their computational method in terms of different validation measures was verified to be reliable and useful to predict potential circRNA-disease associations.

According to a similar method, Wang S. et al. (2019) developed a systematic approach to identify circRNA modules in the BC context through integrating circRNAs, mRNAs, miRNAs, and pathway data based on a non-negative matrix factorization (NMF) algorithm. Employing the systemic pipeline in 33 BC RNA-sequencing data with tumor and normal samples, 13 circRNA modules were identified in BC, containing 80 circRNAs, 2,703 genes, 63 miRNAs, and 1,318 pathways. After screening by functional enrichment analysis, 9 circRNA modules potentially associated with BC were obtained. Within them, one circRNA hsa_circ_0006528 had been recognized as a known disease circRNA. Lei and Fang (2019) proposed a machine learning-based computational model named Gradient Boosting Decision Tree with multiple biological data to predict circRNA-disease associations (GBDTCDA). With this method, the results of those predicting circRNA-disease associations in BC such as circMYO9B and circRNA_100984 were validated to be consistent with the database circ2Disease and circRNADisease. Deng et al. (2019) also established a computational method named KATZCPDA, which was based on the KATZ method and the integrations among circRNAs, proteins, and diseases. By using the KATZCPDA calculation model, BC was ranked at the top of the list of associated diseases. Among the illnesses associated with hsa_circ_0011946, hsa_circ_0001982, hsa_circ_0001785, hsa_circ_0001785, and hsa_circ_0002113, BC was ranked 6th, 6th, 6th, 6th, and 8th, respectively. As detailed in the database, hsa_circ_0001982 had been verified to be involved in the BC cell proliferation and invasion by targeting miR-143.

In 2020, Ge et al. (2020) also invented a computational method named as LLCDC to efficiently predict circRNAs related to BC, showing that 19 of the top 20 potential circRNAs were validated by updating the circRNADisease database and recent biological experiments. Li M. et al. (2020) presented a new method called Speedup Inductive Matrix Completion for CircRNA-Disease Associations prediction (SIMCCDA) to predict circRNA-disease associations. They found that the top 29 predicted candidates were associated with BC in related studies. For instance, hsa_circ_0001875 was upregulated and hsa_circ_0006054 was significantly downregulated in BC tissues (Li M. et al., 2020). Coincidentally, Wang et al. (2020) also proposed a computational method called GCNCDA. In the case study experiments on diseases including BC, glioma, and colorectal cancer, about 16, 15, and 17 of the top 20 candidate circRNAs, respectively, with the highest prediction scores were verified by relevant literature and databases, suggesting that GCNCDA was effective in predicting potential circRNA-disease associations.

These identified circRNA modules provide novel insights into the potentially BC-associated circRNAs. Combining these systematic methods will benefit the clinical applications of circRNA biomarkers for BC diagnosis, treatment, and prognosis in the future.

## CircRNAs in BC Drug Resistance

Currently, chemotherapy plays an indispensable role in the therapy of BC for which it can control and minimize lesions before the operation and prevent recurrence and metastasis after the operation. However, in the process of chemotherapy, patients are prone to drug resistance which hinders the treatment progress. Therefore, it is urgent to slow down or eliminate the chemotherapy resistance of BC and improve the cure rate and quality of life of BC patients. Considering the crucial roles of circRNAs in the chemotherapy resistance of BC therapy, more and more studies are focusing on addressing this obstacle (Table 4). In fact, the resistance-related circRNAs are promising therapeutic and diagnostic biomarkers to increase the chemosensitivity and predict chemotherapeutic agent effectiveness in the individual with resistance thereby improving the clinical management of BC. Adriamycin (ADM), tamoxifen (TAM), and monastrol are the most reported resistant drugs associated with circRNAs in recent studies in the chemotherapy of BC patients.

**Table 4:** The dysregulated circRNAs and their values in breast cancer drug resistance.

**circBase ID (allas)**	**Location**	**Gene symbol**	**Dysregulation**	**miRNA sponge**	**Target gene/pathway**	**Clinical value**	**Reference**
hsa_circ_0006528	chr5:145197456-145205763	PRELID2	Upregulated	miR-7-5p	Raf1 and MAPK/ERK	Promoting ADM resistance of BC	Gao et al., 2017, 2019
hsa_circ_0001839	chr9:6880011-6893232	KDM4C	Downregulated	miR-548p	PBLD	Attenuating ADM resistance and predicting the chemoresponse and prognosis of BC patients	Liang et al., 2019b
hsa_circ_0003218	chr2:203329531-203332412	BMPR2	Downregulated	miR-553	USP4	Mitigating TAM resistance of BC cells	Liang et al., 2019a
hsa_circ_0025202	chr12:6646474-6647162	GAPDH	Downregulated	miR-182-5p	FOXO3a	Exerting TAM sensitization effects and being a therapeutic target in patients with hormone receptor-positive BC receiving TAM therapy	Sang et al., 2019
circ_UBE2D2	–	–	Upregulated	miR-200a-3p	–	Promoting TAM resistance in BC cells	Hu et al., 2020
hsa_circ_0007874	chr6:74175931-74176329	MTO1	Downregulated	–	Eg5	Attenuating monastrol resistance in BC cells.	Liu et al., 2018a

“*–” Not applicable. circRNA, circular RNA; miRNA, microRNA; BC, breast cancer; ADM, adriamycin; TAM, tamoxifen*.

ADM is a cell cycle non-specific antibiotic with a broad antitumor spectrum, which can inhibit the synthesis of DNA and RNA to inhibit tumor growth. ADM is one of the most effective chemotherapy drugs for BC. Nevertheless, the efficacy of ADM is reduced by drug resistance. By circRNA microarray expression profiles and PCR, Gao et al. (2017) found that hsa_circ_0006528 was obviously higher in ADM-resistant human BC tissues and cell lines (MCF-7/ADM and MDA-MB-231/ADM) than that in the ADM-sensitive groups. Additionally, they also discovered a regulatory role of the hsa_circ_0006528-miR-7-5p-Raf1 axis in ADM-resistant BC, strongly inferring that circRNAs participated in BC chemoresistance and further verification and functional analysis were needed (Gao et al., 2019). The circKDM4C was able to regulate BC cell proliferation, metastasis, and ADM resistance both *in vitro* and *in vivo*, mechanistically through sponging miR-548p to increase the PBLD expression (Liang et al., 2019b). CircKDM4C might serve as a biomarker to predict the chemoresponse and prognosis of BC patients, and the approach of targeting the circKDM4C axis might be beneficial for BC treatment (Liang et al., 2019b). Although TAM is a commonly used endocrine therapy for the treatment of advanced BC, TAM resistance remains an inevitable clinical issue for many patients. CircBMPR2 knockdown promoted TAM resistance of BC cells by inhibiting TAM-induced apoptosis, whereas circBMPR2 overexpression led to decreased TAM resistance. Mechanistically, circBMPR2 could inhibit the progression and TAM resistance of BC by sponging miR-553 to upregulate ubiquitin-specific protease 4 (USP4) expression (Liang et al., 2019a). Therefore, circBMPR2 may serve as a potential therapeutic and prognostic predictor for BC patients. Sang et al. (2019) characterized a novel hsa_circ_0025202, which could exert tumor inhibition and TAM sensitization effects *via* the miR-182-5p/FOXO3a axis *in vitro* and *in vivo*. This result confirmed that hsa_circ_0025202 could be a therapeutic target in patients with hormone receptor-positive BC receiving TAM therapy. Additionally, circ_UBE2D2 was upregulated in TAM-resistant BC tissues and cell lines while circ_UBE2D2 deletion mitigated TAM resistance in BC cells (Hu et al., 2020). Circ_UBE2D2 was also significantly loaded in resistant cell-derived exosomes and could be transferred to parental cells. Thus, exosome-mediated transfer of circ_UBE2D2 reinforced the resistance of BC to TAM by binding to miR-200a-3p. Monastrol is the prototype of the anti-actin drug with high efficiency and cell permeability, but its drug resistance limits the application in BC therapy. By using a human circRNA microarray, Liu et al. (2018a) discovered the downregulation of circRNA-MTO1 in monastrol-resistant BC cells. CircRNA-MTO1 could repress Eg5-mediated cellular viability and promote chemosensitivity by sequestering TRAF4 from binding to Eg5 protein. The regulatory pattern of circRNA-MTO1 revealed a regulatory mechanism on circRNA-MTO1 controlling cell viability and monastrol resistance in BC cells. Targeting these newly identified circRNAs may help develop potential novel therapies for BC patients.

## Conclusion

Altogether, the above studies have demonstrated the aberrantly altered expression and biogenesis of circRNAs in different subtypes and stage-specific manners of BC. The fundamental functions of various circRNAs can act as either tumor promoters or suppressors in BC, involving in proliferation, metastasis, and chemotherapy resistance *via* regulating the gene expression (Tran et al., 2020). Most of the circRNA functions in BC are reported to be achieved by miRNA sponge to cause a substantial change in the downstream miRNA activity. These ceRNA functions manifest as circRNA-miRNA-mRNA axis mechanisms (Li et al., 2020). Elucidating these circRNA roles and mechanisms will eventually facilitate the clinical application of circRNAs in the diagnosis, therapy, and prognosis evaluation.

It is worth noting that now this field has still embraced some challenges. Firstly, circRNAs are usually excavated based on RNA sequencing, bioinformatic analyses, and PCR experiments. However, there is a lack of a uniform standard for circRNA naming (Guo et al., 2020). In addition, in some studies, only a small sample size was collected to identify and quantify circRNAs, accompanied by the relatively low abundance of circRNAs, making it possible to acquire the false negatives or inexact expression profiles. Secondly, most of the current researches are still focusing on the exploration of targets and preliminary mechanism verification, but still lacking further mechanism exploration, such as the impact on tumor biogenesis, tumor immunology, and metabolic immune characteristics (Wilusz, 2019). Therefore, more supplementary studies are needed to investigate the molecular mechanisms of their biological functions in BC. Thirdly, generally speaking, the endogenous circRNAs can regulate gene expression *via* binding to miRNAs to inhibit their function in parental cells. It is very important that the circRNA carried by exosomes is shown to transfer to recipient cells, thus playing a gene silencing effect to fine-tune target expression and affecting the environment surrounding the tumor (Shao and Lu, 2020). As exosomes are capable of protecting their cargoes from degradation with high stability in serum and blood, the pluripotent circRNAs encapsulated in exosomes are of more stable peculiarities as biomarkers in BC detection, compared with circRNAs in the cytoplasm. Therefore, exosomal circRNAs are excellent candidates for BC diagnosis and therapy.

Lastly, the circRNAs are of great value in the translation of clinical applications. Because circRNAs are novel ncRNAs, it also emphasizes the clinical significance in diagnosis and treatment. However, most of the existing studies have ascertained the inference and potential of its clinical significance, rather than directly using the target to guide clinical practice (Yin and Liu, 2020). CircRNAs have been recognized as valuable sources of biomarkers and as an auxiliary diagnosis method for the diagnosis and prognosis of BC. Therefore, identifying circRNA disorders in body fluids may be helpful for non-invasive BC diagnosis, but it should be identified and characterized through experiments in the future (Szilágyi et al., 2020). In another aspect, inhibiting overexpressed, pro-tumorigenic circRNAs or sensitizing downregulated, tumor-suppressive circRNAs will be superb strategies for BC therapy. In general, large-scale actual clinical trials in diagnosis and treatment are needed to promote clinical applications of circRNA.

**Figure 3:**
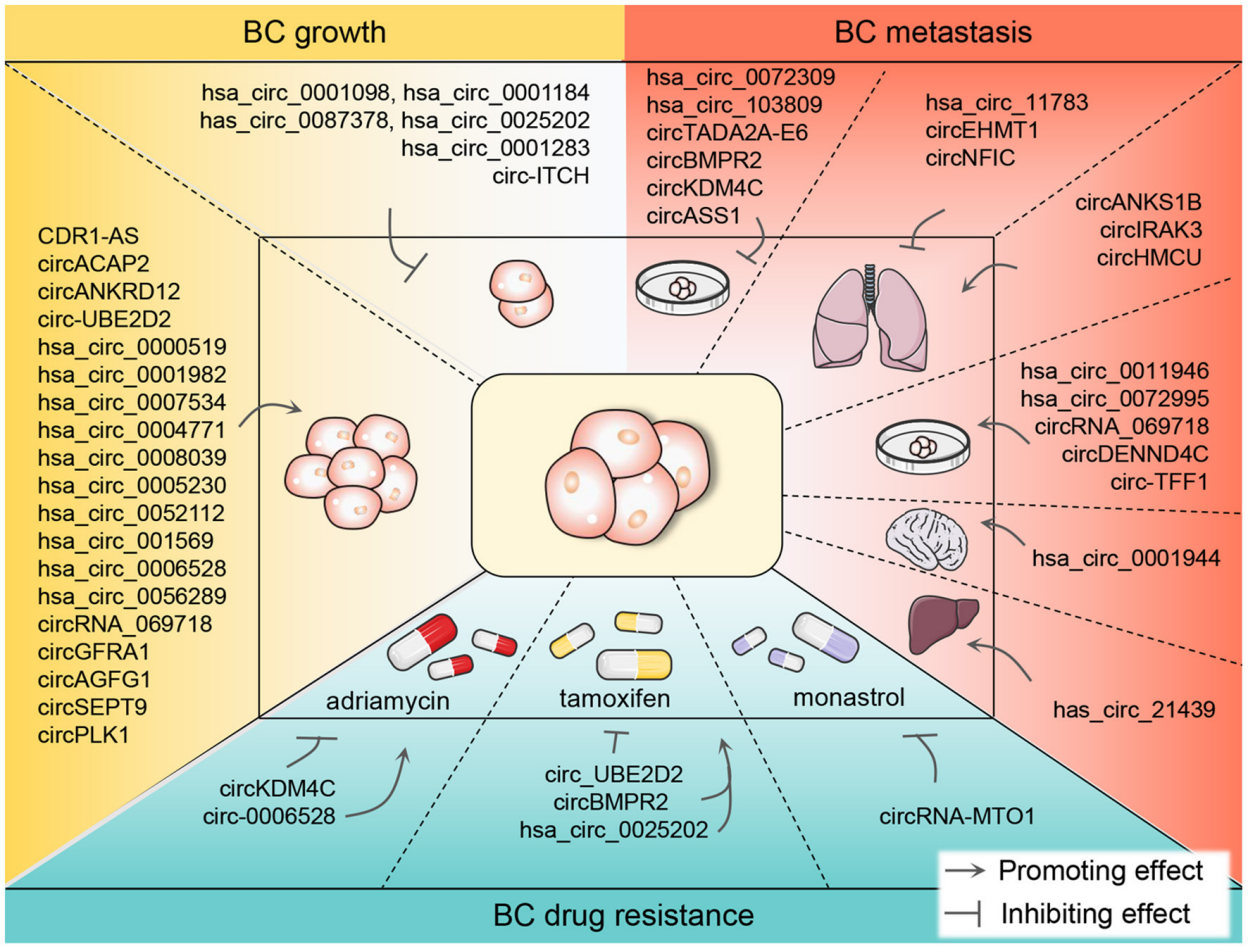
The promoting or inhibiting impacts of differentially expressed circRNAs on the growth, metastasis, and drug resistance of BC. CircRNAs are known to function either as oncogenic or anticancer genes in BC tumor growth and metastasis, promoting BC cell-level metastasis and even the metastasis to the lung, liver, and brain. Currently, chemotherapy plays an indispensable role in the therapy of BC. However, in the process of chemotherapy, patients are prone to drug resistance which hinders the treatment progress. ADM, TAM, and monastrol are the most reported resistant drugs associated with circRNAs in recent studies in the chemotherapy of BC patients. BC, breast cancer; circRNA, circular RNA; ADM, adriamycin; TAM, tamoxifen.

In conclusion, circRNAs are crucial orchestrators in BC growth, metastasis, and drug resistance and are promising biomarkers in BC diagnosis and prognosis (Figure 3). The in-depth elucidation of the relationship between circRNAs and BC will benefit for combating BC.

## Author Contributions

XH, TX, and WH performed the literature search and wrote the manuscript. XL, YWu, and QZ conceived the project and revised the manuscript. YT, DW, YWa, CZ, YY, MX, WL, and MW edited the manuscript. All authors contributed to the article and approved the submitted version.

## Conflict of Interest

The authors declare that the research was conducted in the absence of any commercial or financial relationships that could be construed as a potential conflict of interest.

## Abbreviations

BC: breast cancer
ncRNA: non-coding RNA
lncRNA: long non-coding RNA
circRNA: circular RNA
miRNA: microRNA
ceRNA: competing endogenous RNA
ecircRNA: exonic circRNA
ciRNA: circular intronic RNA
EIciRNA: exon-intronic circRNA
RBP: RNA-binding protein
CDK2: cyclin-dependent kinases 2
snRNA: small nuclear RNA
IRES: internal ribosome entry sites
ORF: open reading frame
ZEB2: zinc finger E-box-binding homeobox 2
TNBC: triple-negative breast cancer
LIF: leukemia inhibitory factor
BCSC: breast cancer stem cell
TCDD: tetrachlorodibenzo-p-dioxin
NF-κB: nuclear factor-κB
ER: estrogen receptor
ECM: extracellular matrix
TNM: tumor-node metastasis
EMT: epithelial-mesenchymal transition
BCBM: breast cancer brain metastases
BCLM: breast cancer liver metastases
TCGA: The Cancer Genome Atlas
qRT-PCR: quantitative real-time polymerase chain reaction
ROR-P: risk-of-relapse proliferation
PWCDA: Path-Weighed Method for Predicting CircRNA-Disease Associations
NMF: non-negative matrix factorization
GBDTCDA: Gradient Boosting Decision Tree with multiple biological data to predict circRNA-disease associations
SIMCCDA: Speedup Inductive Matrix Completion for CircRNA-Disease Associations prediction
USP4: ubiquitin-specific protease 4
ADM: adriamycin
TAM: tamoxifen.

## References

[B1] Barrett S. P. Salzman J. (2016). Circular RNAs: analysis, expression and potential functions. Development 143, 1838–1847. 10.1242/dev.128074 27246710 PMC4920157

[B2] Braicu C. Zimta A.-A. Harangus A. Iurca I. Irimie A. Coza O.. (2019). The function of non-coding RNAs in lung cancer tumorigenesis. Cancers (Basel). 11:605. 10.3390/cancers11050605 31052265 PMC6563001

[B3] Cai F. Fu W. Tang L. Tang J. Sun J. Fu G.. (2020). Hsa_circ_0000515 is a novel circular RNA implicated in the development of breast cancer through its regulation of the microRNA-296-5p/CXCL10 axis. FEBS J. 283, 861–883. 10.1111/febs.15373 32446265

[B4] Cao L. Wang M. Dong Y. Xu B. Chen J. Ding Y.. (2020). Circular RNA circRNF20 promotes breast cancer tumorigenesis and Warburg effect through miR-487a/HIF-1α/HK2. Cell Death Dis. 11:145. 10.1038/s41419-020-2336-0 32094325 PMC7039970

[B5] Chao C. W. Chan D. C. Kuo A. Leder P. (1998). The mouse formin (Fmn) gene: abundant circular RNA transcripts and gene-targeted deletion analysis. Mol. Med. 4, 614–628. 10.1007/BF03401761 9848078 PMC2230310

[B6] Chen B. Wei W. Huang X. Xie X. Kong Y. Dai D.. (2018). circEPSTI1 as a prognostic marker and mediator of triple-negative breast cancer progression. Theranostics 8, 4003–4015. 10.7150/thno.24106 30083277 PMC6071524

[B7] Chen C. Sarnow P. (1995). Initiation of protein synthesis by the eukaryotic translational apparatus on circular RNAs. Science 268, 415–417. 10.1126/science.7536344 7536344

[B8] Chen Y. Li C. Tan C. Liu X. (2016). Circular RNAs: a new frontier in the study of human diseases. J. Med. Genet. 53, 359–365. 10.1136/jmedgenet-2016-103758 26945092

[B9] Deng L. Zhang W. Shi Y. Tang Y. (2019). Fusion of multiple heterogeneous networks for predicting circRNA-disease associations. Sci. Rep. 9:9605. 10.1038/s41598-019-45954-x 31270357 PMC6610109

[B10] Fang S. Pan J. Zhou C. Tian H. He J. Shen W.. (2019). Circular RNAs serve as novel biomarkers and therapeutic targets in cancers. Curr. Gene Ther. 19, 125–133. 10.2174/1566523218666181109142756 30411680

[B11] Fu B. Zhang A. Li M. Pan L. Tang W. An M.. (2018). Circular RNA profile of breast cancer brain metastasis: identification of potential biomarkers and therapeutic targets. Epigenomics 10, 1619–1630. 10.2217/epi-2018-0090 30810051

[B12] Gao D. Qi X. Zhang X. Fang K. Guo Z. Li L. (2019). hsa_circRNA_0006528 as a competing endogenous RNA promotes human breast cancer progression by sponging miR-7-5p and activating the MAPK/ERK signaling pathway. Mol. Carcinog. 58, 554–564. 10.1002/mc.22950 30520151

[B13] Gao D. Zhang X. Liu B. Meng D. Fang K. Guo Z.. (2017). Screening circular RNA related to chemotherapeutic resistance in breast cancer. Epigenomics 9, 1175–1188. 10.2217/epi-2017-0055 28803498

[B14] Ge E. Yang Y. Gang M. Fan C. Zhao Q. (2020). Predicting human disease-associated circRNAs based on locality-constrained linear coding. Genomics 112, 1335–1342. 10.1016/j.ygeno.2019.08.001 31394170

[B15] Geng Y. Jiang J. Wu C. (2018). Function and clinical significance of circRNAs in solid tumors. J. Hematol. Oncol. 11:98. 10.1186/s13045-018-0643-z 30064463 PMC6069963

[B16] Greene J. Baird A.-M. Brady L. Lim M. Gray S. G. McDermott R.. (2017). Circular RNAs: biogenesis, function and role in human diseases. Front. Mol. Biosci. 8:38. 10.3389/fmolb.2017.00038 28634583 PMC5459888

[B17] Gu Y. Ci C. Zhang X. Su M. Lv W. Chen C.. (2019). Prediction of circRNAs based on the DNA methylation-mediated feature sponge function in breast cancer. Front. Bioeng. Biotechnol. 7:365. 10.3389/fbioe.2019.00365 32039169 PMC6988805

[B18] Guo Z. Cao Q. Zhao Z. Song C. (2020). Biogenesis, features, functions, and disease relationships of a specific circular RNA: CDR1as. Aging Dis. 11, 1009–1020. 10.14336/AD.2019.0920 32765960 PMC7390531

[B19] Han T.-S. Hur K. Cho H.-S. Ban H. S. (2020). Epigenetic associations between lncRNA/circRNA and miRNA in hepatocellular carcinoma. Cancers (Basel). 12:2622. 10.3390/cancers12092622 32937886 PMC7565033

[B20] Hansen T. B. Jensen T. I. Clausen B. H. Bramsen J. B. Finsen B. Damgaard C. K.. (2013). Natural RNA circles function as efficient microRNA sponges. Nature 495, 384–388. 10.1038/nature11993 23446346

[B21] He R. Liu P. Xie X. Zhou Y. Liao Q. Xiong W.. (2017). circGFRA1 and GFRA1 act as ceRNAs in triple negative breast cancer by regulating miR-34a. J. Exp. Clin. Cancer Res. 36:145. 10.1186/s13046-017-0614-1 29037220 PMC5644184

[B22] Hou J. Xu Z. Zhong S. Zhang H. Jiang L. Chen X.. (2019). Circular RNA circASS1 is downregulated in breast cancer cells MDA-MB-231 and suppressed invasion and migration. Epigenomics 11, 199–213. 10.2217/epi-2017-0167 30657346

[B23] Hu K. Liu X. Li Y. Li Q. Xu Y. Zeng W.. (2020). Exosomes mediated transfer of Circ_UBE2D2 enhances the resistance of breast cancer to tamoxifen by binding to MiR-200a-3p. Med. Sci. Monit. 26:e922253. 10.12659/MSM.922253 32756532 PMC7431386

[B24] Hu Y. Guo F. Zhu H. Tan X. Zhu X. Liu X.. (2020). Circular RNA-0001283 suppresses breast cancer proliferation and invasion via MiR-187/HIPK3 axis. Med. Sci. Monit. 26:e921502. 10.12659/MSM.921502 32066649 PMC7047918

[B25] Jeck W. R. Sorrentino J. A. Wang K. Slevin M. K. Burd C. E. Liu J.. (2013). Circular RNAs are abundant, conserved, and associated with ALU repeats. RNA 19, 141–157. 10.1261/rna.035667.112 23249747 PMC3543092

[B26] Jiang W. Rixiati Y. Huang H. Shi Y. Huang C. Jiao B. (2020). Asperolide A prevents bone metastatic breast cancer via the PI3K/AKT/mTOR/c-Fos/NFATc1 signaling pathway. Cancer Med. 9, 8173–8185. 10.1002/cam4.3432 32976685 PMC7643645

[B27] Kalyana-Sundaram S. Kumar-Sinha C. Shankar S. Robinson D. R. Wu Y.-M. Cao X.. (2012). Expressed pseudogenes in the transcriptional landscape of human cancers. Cell 149, 1622–1634. 10.1016/j.cell.2012.04.041 22726445 PMC3597446

[B28] Karedath T. Ahmed I. Al Ameri W. Al-Dasim F. M. Andrews S. S. Samuel S.. (2019). Silencing of ANKRD12 circRNA induces molecular and functional changes associated with invasive phenotypes. BMC Cancer 19:565. 10.1186/s12885-019-5723-0 31185953 PMC6558796

[B29] Kong Y. Yang L. Wei W. Lyu N. Zou Y. Gao G.. (2019). circPLK1 sponges miR-296-5p to facilitate triple-negative breast cancer progression. Epigenomics 11, 1163–1176. 10.2217/epi-2019-0093 31337246

[B30] Legnini I. Di Timoteo G. Rossi F. Morlando M. Briganti F. Sthandier O.. (2017). circ-ZNF609 is a circular RNA that can be translated and functions in myogenesis. Mol. Cell 66, 22.e9–37.e9. 10.1016/j.molcel.2017.02.017 28344082 PMC5387670

[B31] Lei X. Fang Z. (2019). GBDTCDA: predicting circRNA-disease associations based on gradient boosting decision tree with multiple biological data fusion. Int. J. Biol. Sci. 15, 2911–2924. 10.7150/ijbs.33806 31853227 PMC6909967

[B32] Lei X. Fang Z. Chen L. Wu F.-X. (2018). PWCDA: path weighted method for predicting circRNA-disease associations. Int. J. Mol. Sci. 19:3410. 10.3390/ijms19113410 30384427 PMC6274797

[B33] Li L.-J. Leng R.-X. Fan Y.-G. Pan H.-F. Ye D.-Q. (2017). Translation of noncoding RNAs: focus on lncRNAs, pri-miRNAs, and circRNAs. Exp. Cell Res. 361, 1–8. 10.1016/j.yexcr.2017.10.010 29031633

[B34] Li M. Liu M. Bin Y. Xia J. (2020). Prediction of circRNA-disease associations based on inductive matrix completion. BMC Med. Genomics 13:42. 10.1186/s12920-020-0679-0 32241268 PMC7118830

[B35] Li W. Yang X. Shi C. Zhou Z. (2020). Hsa_circ_002178 promotes the growth and migration of breast cancer cells and maintains cancer stem-like cell properties through regulating miR-1258/KDM7A axis. Cell Transplant. 29:096368972096017. 10.1177/0963689720960174 32951449 PMC7784609

[B36] Li Z. Chen Z. Hu G. Zhang Y. Feng Y. Jiang Y.. (2020). Profiling and integrated analysis of differentially expressed circRNAs as novel biomarkers for breast cancer. J. Cell. Physiol. 235, 7945–7959. 10.1002/jcp.29449 31943203

[B37] Li Z. Ruan Y. Zhang H. Shen Y. Li T. Xiao B. (2019). Tumor-suppressive circular RNAs: mechanisms underlying their suppression of tumor occurrence and use as therapeutic targets. Cancer Sci. 110, 3630–3638. 10.1111/cas.14211 31599076 PMC6890437

[B38] Liang G. Ling Y. Mehrpour M. Saw P. E. Liu Z. Tan W.. (2020). Autophagy-associated circRNA circCDYL augments autophagy and promotes breast cancer progression. Mol. Cancer 19:65. 10.1186/s12943-020-01152-2 32213200 PMC7093993

[B39] Liang Y. Song X. Li Y. Ma T. Su P. Guo R.. (2019a). Targeting the circBMPR2/miR-553/USP4 axis as a potent therapeutic approach for breast cancer. Mol. Ther. Nucleic Acids 17, 347–361. 10.1016/j.omtn.2019.05.005 31302495 PMC6626870

[B40] Liang Y. Song X. Li Y. Su P. Han D. Ma T.. (2019b). circKDM4C suppresses tumor progression and attenuates doxorubicin resistance by regulating miR-548p/PBLD axis in breast cancer. Oncogene 38, 6850–6866. 10.1038/s41388-019-0926-z 31406252

[B41] Lin X. Hong S. Chen J. Chen W. Wu Z. (2019). The potential targets for metastases: a study on altered circular RNA profile in breast cancer liver metastases. Epigenomics 11, 1237–1250. 10.2217/epi-2019-0099 31293174

[B42] Liu M. Luo C. Dong J. Guo J. Luo Q. Ye C.. (2020). circRNA_103809 suppresses the proliferation and metastasis of breast cancer cells by sponging microRNA-532-3p (miR-532-3p). Front. Genet. 11:485. 10.3389/fgene.2020.00485 32499818 PMC7243809

[B43] Liu P. Zou Y. Li X. Yang A. Ye F. Zhang J.. (2020). circGNB1 facilitates triple-negative breast cancer progression by regulating miR-141-5p-IGF1R Axis. Front. Genet. 11:193. 10.3389/fgene.2020.00193 32194644 PMC7066119

[B44] Liu T. Ye P. Ye Y. Lu S. Han B. (2020). Circular RNA hsa_circRNA_002178 silencing retards breast cancer progression via microRNA-328-3p-mediated inhibition of COL1A1. J. Cell. Mol. Med. 24, 2189–2201. 10.1111/jcmm.14875 31957232 PMC7011152

[B45] Liu Y. Dong Y. Zhao L. Su L. Luo J. (2018a). Circular RNA-MTO1 suppresses breast cancer cell viability and reverses monastrol resistance through regulating the TRAF4/Eg5 axis. Int. J. Oncol. 53, 1752–1762. 10.3892/ijo.2018.4485 30015883

[B46] Liu Y. Lu C. Zhou Y. Zhang Z. Sun L. (2018b). Circular RNA hsa_circ_0008039 promotes breast cancer cell proliferation and migration by regulating miR-432-5p/E2F3 axis. Biochem. Biophys. Res. Commun. 502, 358–363. 10.1016/j.bbrc.2018.05.166 29807010

[B47] Liu Z. Zhou Y. Liang G. Ling Y. Tan W. Tan L.. (2019). Circular RNA hsa_circ_001783 regulates breast cancer progression via sponging miR-200c-3p. Cell Death Dis. 10:55. 10.1038/s41419-018-1287-1 30670688 PMC6343010

[B48] Lou W. Ding B. Fu P. (2020). Pseudogene-derived lncRNAs and their miRNA sponging mechanism in human cancer. Front. Cell Dev. Biol. 8:85. 10.3389/fcell.2020.00085 32185172 PMC7058547

[B49] Lu M. Wu Y. Zeng B. Sun J. Li Y. Luo J.. (2020). circEHMT1 inhibits metastatic potential of breast cancer cells by modulating miR-1233-3p/KLF4/MMP2 axis. Biochem. Biophys. Res. Commun. 526, 306–313. 10.1016/j.bbrc.2020.03.084 32209259

[B50] Ma X. Liu C. Gao C. Li J. Zhuang J. Liu L.. (2020). circRNA-associated ceRNA network construction reveals the circRNAs involved in the progression and prognosis of breast cancer. J. Cell. Physiol. 235, 3973–3983. 10.1002/jcp.29291 31617204 PMC13482726

[B51] Memczak S. Jens M. Elefsinioti A. Torti F. Krueger J. Rybak A.. (2013). Circular RNAs are a large class of animal RNAs with regulatory potency. Nature 495, 333–338. 10.1038/nature11928 23446348

[B52] Nair A. A. Niu N. Tang X. Thompson K. J. Wang L. Kocher J.-P.. (2016). Circular RNAs and their associations with breast cancer subtypes. Oncotarget 7, 80967–80979. 10.18632/oncotarget.13134 27829232 PMC5348369

[B53] Nedoluzhko A. Sharko F. Rbbani M. G. Teslyuk A. Konstantinidis I. Fernandes J. M. O. (2020). CircParser: a novel streamlined pipeline for circular RNA structure and host gene prediction in non-model organisms. PeerJ 8:e8757. 10.7717/peerj.8757 32211235 PMC7081776

[B54] Pan G. Mao A. Liu J. Lu J. Ding J. Liu W. (2020). Circular RNA hsa_circ_0061825 (circ-TFF1) contributes to breast cancer progression through targeting miR-326/TFF1 signalling. Cell Prolif. 53, 1–14. 10.1111/cpr.12720 31961997 PMC7048212

[B55] Pan H. Li T. Jiang Y. Pan C. Ding Y. Huang Z.. (2018). Overexpression of circular RNA ciRS-7 abrogates the tumor suppressive effect of miR-7 on gastric cancer via PTEN/PI3K/AKT signaling pathway. J. Cell. Biochem. 119, 440–446. 10.1002/jcb.26201 28608528

[B56] Qu S. Yang X. Li X. Wang J. Gao Y. Shang R.. (2015). Circular RNA: a new star of noncoding RNAs. Cancer Lett. 365, 141–148. 10.1016/j.canlet.2015.06.003 26052092

[B57] Ren S. Liu J. Feng Y. Li Z. He L. Li L.. (2019). Knockdown of circDENND4C inhibits glycolysis, migration and invasion by up-regulating miR-200b/c in breast cancer under hypoxia. J. Exp. Clin. Cancer Res. 38:388. 10.1186/s13046-019-1398-2 31488193 PMC6727545

[B58] Ruan Y. Li Z. Shen Y. Li T. Zhang H. Guo J. (2020). Functions of circular RNAs and their potential applications in gastric cancer. Expert Rev. Gastroenterol. Hepatol. 14, 85–92. 10.1080/17474124.2020.1715211 31922886

[B59] Sang Y. Chen B. Song X. Li Y. Liang Y. Han D.. (2019). circRNA_0025202 regulates tamoxifen sensitivity and tumor progression via regulating the miR-182-5p/FOXO3a axis in breast cancer. Mol. Ther. 27, 1638–1652. 10.1016/j.ymthe.2019.05.011 31153828 PMC6731174

[B60] Sanger H. L. Klotz G. Riesner D. Gross H. J. Kleinschmidt A. K. (1976). Viroids are single-stranded covalently closed circular RNA molecules existing as highly base-paired rod-like structures. Proc. Natl. Acad. Sci. U.S.A. 73, 3852–3856. 10.1073/pnas.73.11.3852 1069269 PMC431239

[B61] Shao Y. Lu B. (2020). The crosstalk between circular RNAs and the tumor microenvironment in cancer metastasis. Cancer Cell Int. 20:448. 10.1186/s12935-020-01532-0 32943996 PMC7488731

[B62] Shi P. Sun J. He B. Song H. Li Z. Kong W.. (2018). Profiles of differentially expressed circRNAs in esophageal and breast cancer. Cancer Manag. Res. 10, 2207–2221. 10.2147/CMAR.S167863 30087579 PMC6061203

[B63] Smid M. Wilting S. M. Uhr K. Rodríguez-González F. G. de Weerd V. Prager-Van der Smissen W. J. C.. (2019). The circular RNome of primary breast cancer. Genome Res. 29, 356–366. 10.1101/gr.238121.118 30692147 PMC6396421

[B64] Song L. Xiao Y. (2018). Downregulation of hsa_circ_0007534 suppresses breast cancer cell proliferation and invasion by targeting miR-593/MUC19 signal pathway. Biochem. Biophys. Res. Commun. 503, 2603–2610. 10.1016/j.bbrc.2018.08.007 30139516

[B65] Song X. Liang Y. Sang Y. Li Y. Zhang H. Chen B.. (2020). circHMCU promotes proliferation and metastasis of breast cancer by sponging the let-7 family. Mol. Ther. Nucleic Acids 20, 518–533. 10.1016/j.omtn.2020.03.014 32330870 PMC7178009

[B66] Sun Z. Chen C. Su Y. Wang W. Yang S. Zhou Q.. (2019). Regulatory mechanisms and clinical perspectives of circRNA in digestive system neoplasms. J. Cancer 10, 2885–2891. 10.7150/jca.31167 31281465 PMC6590048

[B67] Szilágyi M. Pös O. Márton É. Buglyó G. Soltész B. Keseru J.. (2020). Circulating cell-free nucleic acids: main characteristics and clinical application. Int. J. Mol. Sci. 21:6827. 10.3390/ijms21186827 32957662 PMC7555669

[B68] Tang H. Huang X. Wang J. Yang L. Kong Y. Gao G.. (2019). circKIF4A acts as a prognostic factor and mediator to regulate the progression of triple-negative breast cancer. Mol. Cancer 18:23. 10.1186/s12943-019-0946-x 30744636 PMC6369546

[B69] Tang Y.-Y. Zhao P. Zou T.-N. Duan J.-J. Zhi R. Yang S.-Y.. (2017). Circular RNA hsa_circ_0001982 promotes breast cancer cell carcinogenesis through decreasing miR-143. DNA Cell Biol. 36, 901–908. 10.1089/dna.2017.3862 28933584

[B70] Tarrero L. C. Ferrero G. Miano V. De Intinis C. Ricci L. Arigoni M.. (2018). Luminal breast cancer-specific circular RNAs uncovered by a novel tool for data analysis. Oncotarget 9, 14580–14596. 10.18632/oncotarget.24522 29581865 PMC5865691

[B71] Tomar D. Yadav A. S. Kumar D. Bhadauriya G. Kundu G. C. (2020). Non-coding RNAs as potential therapeutic targets in breast cancer. Biochim. Biophys. Acta Gene Regul. Mech. 1863:194378. 10.1016/j.bbagrm.2019.04.005 31048026

[B72] Tran A. M. Chalbatani G. M. Berland L. Cruz De los Santos M. Raj P. Jalali S. A.. (2020). A new world of biomarkers and therapeutics for female reproductive system and breast cancers: circular RNAs. Front. Cell Dev. Biol. 8:50. 10.3389/fcell.2020.00050 32211400 PMC7075436

[B73] Trimboli R. M. Giorgi Rossi P. Battisti N. M. L. Cozzi A. Magni V. Zanardo M.. (2020). Do we still need breast cancer screening in the era of targeted therapies and precision medicine? Insights Imaging 11:105. 10.1186/s13244-020-00905-3 32975658 PMC7519022

[B74] Uhr K. Sieuwerts A. M. de Weerd V. Smid M. Hammerl D. Foekens J. A.. (2018). Association of microRNA-7 and its binding partner CDR1-AS with the prognosis and prediction of 1st-line tamoxifen therapy in breast cancer. Sci. Rep. 8:9657. 10.1038/s41598-018-27987-w 29941867 PMC6018428

[B75] Venø M. T. Hansen T. B. Venø S. T. Clausen B. H. Grebing M. Finsen B.. (2015). Spatio-temporal regulation of circular RNA expression during porcine embryonic brain development. Genome Biol. 16:245. 10.1186/s13059-015-0801-3 26541409 PMC4635978

[B76] Wang H. Xiao Y. Wu L. Ma D. (2018). Comprehensive circular RNA profiling reveals the regulatory role of the circRNA-000911/miR-449a pathway in breast carcinogenesis. Int. J. Oncol. 52, 743–754. 10.3892/ijo.2018.4265 29431182 PMC5807038

[B77] Wang J. Zhang Q. Zhou S. Xu H. Wang D. Feng J.. (2019). Circular RNA expression in exosomes derived from breast cancer cells and patients. Epigenomics 11, 411–421. 10.2217/epi-2018-0111 30785332

[B78] Wang L. You Z.-H. Li Y.-M. Zheng K. Huang Y.-A. (2020). GCNCDA: a new method for predicting circRNA-disease associations based on Graph Convolutional Network Algorithm. PLOS Comput. Biol. 16:e1007568. 10.1371/journal.pcbi.1007568 32433655 PMC7266350

[B79] Wang Q. Li Z. Hu Y. Zheng W. Tang W. Zhai C.. (2019). circ-TFCP2L1 promotes the proliferation and migration of triple negative breast cancer through sponging miR-7 by inhibiting PAK1. J. Mammary Gland Biol. Neoplasia 24, 323–331. 10.1007/s10911-019-09440-4 31776835

[B80] Wang S. Li Q. Wang Y. Li X. Wang R. Kang Y.. (2018). Upregulation of circ-UBAP2 predicts poor prognosis and promotes triple-negative breast cancer progression through the miR-661/MTA1 pathway. Biochem. Biophys. Res. Commun. 505, 996–1002. 10.1016/j.bbrc.2018.10.026 30314706

[B81] Wang S. Xia P. Zhang L. Yu L. Liu H. Meng Q.. (2019). Systematical identification of breast cancer-related circular RNA modules for deciphering circRNA functions based on the non-negative matrix factorization algorithm. Int. J. Mol. Sci. 20:919. 10.3390/ijms20040919 30791568 PMC6412941

[B82] Wang S. T. Liu L. B. Li X. M. Wang Y. F. Xie P. J. Li Q.. (2019). circ-ITCH regulates triple-negative breast cancer progression through the Wnt/β-catenin pathway. Neoplasma 66, 232–239. 10.4149/neo_2018_180710N460 30509108

[B83] Wang Y. Li J. Du C. Zhang L. Zhang Y. Zhang J.. (2019). Upregulated circular RNA circ-UBE2D2 predicts poor prognosis and promotes breast cancer progression by sponging miR-1236 and miR-1287. Transl. Oncol. 12, 1305–1313. 10.1016/j.tranon.2019.05.016 31336316 PMC6657235

[B84] Wang Y. Mo Y. Gong Z. Yang X. Yang M. Zhang S.. (2017). Circular RNAs in human cancer. Mol. Cancer 16:25. 10.1186/s12943-017-0598-7 28143578 PMC5282898

[B85] Wilusz J. E. (2019). Circle the wagons: circular RNAs control innate immunity. Cell 177, 797–799. 10.1016/j.cell.2019.04.020 31051101 PMC7112311

[B86] Wu J. Cheng J. Zhang F. Luo X. Zhang Z. Chen S. (2020). Estrogen receptor α is involved in the regulation of ITGA8 methylation in estrogen receptor-positive breast cancer. Ann. Transl. Med. 8, 993–993. 10.21037/atm-20-5220 32953793 PMC7475494

[B87] Wu J. Jiang Z. Chen C. Hu Q. Fu Z. Chen J.. (2018). circIRAK3 sponges miR-3607 to facilitate breast cancer metastasis. Cancer Lett. 430, 179–192. 10.1016/j.canlet.2018.05.033 29803789

[B88] Xiao W. Zheng S. Zou Y. Yang A. Xie X. Tang H.. (2019). circAHNAK1 inhibits proliferation and metastasis of triple-negative breast cancer by modulating miR-421 and RASA1. Aging (Albany. NY). 11, 12043–12056. 10.18632/aging.102539 31857500 PMC6949091

[B89] Xie R. Tang J. Zhu X. Jiang H. (2019). Silencing of hsa_circ_0004771 inhibits proliferation and induces apoptosis in breast cancer through activation of miR-653 by targeting ZEB2 signaling pathway. Biosci. Rep. 39:BSR20181919. 10.1042/BSR20181919 30979827 PMC6522819

[B90] Xie R. Zhang Y. Zhang J. Li J. Zhou X. (2020). The role of circular RNAs in immune-related diseases. Front. Immunol. 11:545. 10.3389/fimmu.2020.00545 32300345 PMC7142234

[B91] Xing L. Yang R. Wang X. Zheng X. Yang X. Zhang L.. (2020). The circRNA circIFI30 promotes progression of triple-negative breast cancer and correlates with prognosis. Aging (Albany. NY). 12, 10983–11003. 10.18632/aging.103311 32497020 PMC7346060

[B92] Xu G. Ye D. Zhao Q. He R. Ma W. Li Y.. (2020). circNFIC suppresses breast cancer progression by sponging miR-658. J. Cancer 11, 4222–4229. 10.7150/jca.38830 32368305 PMC7196272

[B93] Xu J. Wang Y. Xu D. (2019). Hsa_circ_001569 is an unfavorable prognostic factor and promotes cell proliferation and metastasis by modulating PI3K-AKT pathway in breast cancer. Cancer Biomarkers 25, 193–201. 10.3233/CBM-182293 31104012 PMC13082401

[B94] Xu J. Wu K. Jia Q. Ding X. (2020). Roles of miRNA and IncRNA in triple-negative breast cancer. J. Zhejiang Univ. B 21, 673–689. 10.1631/jzus.B1900709 32893525 PMC7519626

[B95] Xu J.-Z. Shao C.-C. Wang X.-J. Zhao X. Chen J.-Q. Ouyang Y.-X.. (2019). circTADA2As suppress breast cancer progression and metastasis via targeting miR-203a-3p/SOCS3 axis. Cell Death Dis. 10:175. 10.1038/s41419-019-1382-y 30787278 PMC6382814

[B96] Xu Y. Yao Y. Leng K. Ji D. Qu L. Liu Y.. (2018). Increased expression of circular RNA circ_0005230 indicates dismal prognosis in breast cancer and regulates cell proliferation and invasion via miR-618/CBX8 signal pathway. Cell. Physiol. Biochem. 51, 1710–1722. 10.1159/000495675 30504704

[B97] Yan L. Zheng M. Wang H. (2019). Circular RNA hsa_circ_0072309 inhibits proliferation and invasion of breast cancer cells via targeting miR-492. Cancer Manag. Res. 11, 1033–1041. 10.2147/CMAR.S186857 30774431 PMC6349082

[B98] Yan N. Xu H. Zhang J. Xu L. Zhang Y. Zhang L.. (2017). Circular RNA profile indicates circular RNA VRK1 is negatively related with breast cancer stem cells. Oncotarget 8, 95704–95718. 10.18632/oncotarget.21183 29221160 PMC5707054

[B99] Yang L. Song C. Chen Y. Jing G. Sun J. (2019). Circular RNA circ_0103552 forecasts dismal prognosis and promotes breast cancer cell proliferation and invasion by sponging miR-1236. J. Cell. Biochem. 120, 15553–15560. 10.1002/jcb.28822 31056795

[B100] Yang R. Xing L. Zheng X. Sun Y. Wang X. Chen J. (2019). The circRNA circAGFG1 acts as a sponge of miR-195-5p to promote triple-negative breast cancer progression through regulating CCNE1 expression. Mol. Cancer 18:4. 10.1186/s12943-018-0933-7 30621700 PMC6325825

[B101] Yang S. Wang D. Zhou S. Zhang Q. Wang J. Zhong S.. (2020). Identification of circRNA–miRNA networks for exploring an underlying prognosis strategy for breast cancer. Epigenomics 12, 101–125. 10.2217/epi-2019-0058 31920098

[B102] Yin K. Liu X. (2020). circMMP1 promotes the progression of glioma through miR-433/HMGB3 axis *in vitro* and *in vivo*. IUBMB Life 72, 2508–2524. 10.1002/iub.2383 32918539

[B103] Yuan C. Zhou L. Zhang L. Yin K. Peng J. Sha R.. (2019). Identification and integrated analysis of key differentially expressed circular RNAs in ER-positive subtype breast cancer. Epigenomics 11, 297–321. 10.2217/epi-2018-0147 30417652

[B104] Zeng K. He B. Yang B. B. Xu T. Chen X. Xu M.. (2018). The pro-metastasis effect of circANKS1B in breast cancer. Mol. Cancer 17:160. 10.1186/s12943-018-0914-x 30454010 PMC6240936

[B105] Zhang C. Wu H. Wang Y. Zhao Y. Fang X. Chen C.. (2015). Expression patterns of circular RNAs from primary kinase transcripts in the mammary glands of lactating rats. J. Breast Cancer 18:235. 10.4048/jbc.2015.18.3.235 26472973 PMC4600687

[B106] Zhang H. Jiang L. Hou J. Zhong S. Zhou S. Zhu L.. (2018a). Circular RNA hsa_circ_0052112 promotes cell migration and invasion by acting as sponge for miR-125a-5p in breast cancer. Biomed. Pharmacother. 107, 1342–1353. 10.1016/j.biopha.2018.08.030 30257349

[B107] Zhang H.-D. Jiang L.-H. Hou J.-C. Zhou S.-Y. Zhong S.-L. Zhu L.-P.. (2018b). Circular RNA hsa_circ_0072995 promotes breast cancer cell migration and invasion through sponge for miR-30c-2-3p. Epigenomics 10, 1229–1242. 10.2217/epi-2018-0002 30182731

[B108] Zhang J. Xu H. D. Xing X. J. Liang Z. T. Xia Z. H. Zhao Y. (2019). circRNA-069718 promotes cell proliferation and invasion in triple-negative breast cancer by activating Wnt/β-catenin pathway. Eur. Rev. Med. Pharmacol. Sci. 23, 5315–5322. 10.26355/eurrev_201906_18198 31298383

[B109] Zhang M. Huang N. Yang X. Luo J. Yan S. Xiao F.. (2018). A novel protein encoded by the circular form of the SHPRH gene suppresses glioma tumorigenesis. Oncogene 37, 1805–1814. 10.1038/s41388-017-0019-9 29343848

[B110] Zhang Y. Zhang X.-O. Chen T. Xiang J.-F. Yin Q.-F. Xing Y.-H.. (2013). Circular intronic long noncoding RNAs. Mol. Cell 51, 792–806. 10.1016/j.molcel.2013.08.017 24035497

[B111] Zhao B. Song X. Guan H. (2020). circACAP2 promotes breast cancer proliferation and metastasis by targeting miR-29a/b-3p-COL5A1 axis. Life Sci. 244, 117179. 10.1016/j.lfs.2019.117179 31863774

[B112] Zhao C.-H. Qu L. Zhang H. Qu R. (2019). Identification of breast cancer-related circRNAs by analysis of microarray and RNA-sequencing data. Medicine (Baltimore). 98:e18042. 10.1097/MD.0000000000018042 31725681 PMC6867785

[B113] Zhao J. Zou H. Han C. Ma J. Zhao J. Tang J. (2018). Circlular RNA BARD1 (Hsa_circ_0001098) overexpression in breast cancer cells with TCDD treatment could promote cell apoptosis via miR-3942/BARD1 axis. Cell Cycle 17, 2731–2744. 10.1080/15384101.2018.1556058 30521417 PMC6343738

[B114] Zheng X. Huang M. Xing L. Yang R. Wang X. Jiang R.. (2020). The circRNA circSEPT9 mediated by E2F1 and EIF4A3 facilitates the carcinogenesis and development of triple-negative breast cancer. Mol. Cancer 19:73. 10.1186/s12943-020-01183-9 32264877 PMC7137343

[B115] Zhou J. Zhang W.-W. Peng F. Sun J.-Y. He Z.-Y. Wu S.-G. (2018). Downregulation of hsa_circ_0011946 suppresses the migration and invasion of the breast cancer cell line MCF-7 by targeting RFC3. Cancer Manag. Res. 10, 535–544. 10.2147/CMAR.S155923 29593432 PMC5865555

[B116] Zhou S. Chen W. Yang S. Xu Z. Hu J. Zhang H.. (2019). The emerging role of circular RNAs in breast cancer. Biosci. Rep. 39:BSR20190621. 10.1042/BSR20190621 31160488 PMC6591565

